# Deciphering clock cell network morphology within the biological master clock, suprachiasmatic nucleus: From the perspective of circadian wave dynamics

**DOI:** 10.1371/journal.pcbi.1010213

**Published:** 2022-06-06

**Authors:** Hyun Kim, Cheolhong Min, Byeongha Jeong, Kyoung J. Lee

**Affiliations:** 1 Department of Physics, Korea University, Seoul, Korea; 2 University of Texas Southwestern Medical Center, Dallas, Texas, United States of America; University of Cincinnati College of Medicine, UNITED STATES

## Abstract

The biological master clock, suprachiasmatic nucleus (of rat and mouse), is composed of ~10,000 clock cells which are heterogeneous with respect to their circadian periods. Despite this inhomogeneity, an intact SCN maintains a very good degree of circadian phase (time) coherence which is vital for sustaining various circadian rhythmic activities, and it is supposedly achieved by not just one but a few different cell-to-cell coupling mechanisms, among which action potential (AP)-mediated connectivity is known to be essential. But, due to technical difficulties and limitations in experiments, so far very little information is available about the morphology of the connectivity at a cellular scale. Building upon this limited amount of information, here we exhaustively and systematically explore a large pool (~25,000) of various network morphologies to come up with some plausible network features of SCN networks. All candidates under consideration reflect an experimentally obtained ‘indegree distribution’ as well as a ‘physical range distribution of afferent clock cells.’ Then, importantly, with a set of multitude criteria based on the properties of SCN circadian phase waves in extrinsically perturbed as well as in their natural states, we select out appropriate model networks: Some important measures are, 1) level of phase dispersal and direction of wave propagation, 2) phase-resetting ability of the model networks subject to external circadian forcing, and 3) decay rate of perturbation induced “phase-singularities.” The successful, realistic networks have several common features: 1) “indegree” and “outdegree” should have a positive correlation; 2) the cells in the SCN ventrolateral region (core) have a much larger total degree than that of the dorsal medial region (shell); 3) The number of intra-core edges is about 7.5 times that of intra-shell edges; and 4) the distance probability density function for the afferent connections fits well to a beta function. We believe that these newly identified network features would be a useful guide for future explorations on the very much unknown AP-mediated clock cell connectome within the SCN.

## Author summary

The suprachiasmatic nucleus (SCN) is a tiny nucleus located within a deep brain area, serving the role of the biological master clock for mammals. It is composed of approximately 10,000 clock cells and orchestrates all daily rhythms such as the sleep-wake cycle and heart-beat rate modulation. The SCN is a vitally important part of the brain. Any significant disruptions in the spatiotemporal circadian dynamics inside this small nucleus may potentially lead to circadian arrhythmia akin to cardiac arrhythmia. One of the key elements governing the spatiotemporal circadian dynamics is the SCN clock cell-to-cell network morphology, which is currently largely unknown and very difficult to decipher even with all up-to-date technologies in neuroscience. Therefore, the purpose of this paper is to come up with a mathematical model SCN that has a clock cell network morphology good enough that the model SCN’s spatiotemporal phase dynamics during perturbed as well as unperturbed natural states best matches those measured in experiments. We find many interesting features, some of which are 1) the outdegree (number of efferent connections) of each clock cell should have a positive correlation to its indegree (number of afferent connections), 2) the total degree of the cells in the SCN ‘core’ region is in general much larger than that of the ‘shell’ region, 3) the number of intra-core edges is about 7.5 times that of intra-shell edges; and 4) the number of inter-subdivisional edges should be much less than that of intra-core edges. These features identified via model simulations should be a useful guide for future experimental explorations on the clock cell connectome within the SCN.

## Introduction

It is now well established that rat (or mouse) SCN is a pair of two small nuclei of ~10,000 clock cells [1, 2], which are either a self-sustained circadian oscillator or a damped oscillator that can be entrained to other oscillators [3, 4]. The autonomously oscillating clock cells are heterogeneous with respect to their circadian periods (ranging 20 ~ 28 hours) [5] as well as biological markers (e.g., VIP, AVP, or GRP) which they release [6]. Despite these heterogeneities, however, within intact (or even in organotypic slice cultures of) SCNs the circadian oscillators not only synchronize themselves (i.e., frequency-locked) but also maintain a good degree of circadian phase coherence, which is generally rendered visible as a systemwide, large-scale, propagating phase wave. Maintaining a good phase coherence within the SCN is vital for mammals sustaining various circadian rhythmic activities during mundane life; one can imagine that any significant disruptions in the spatiotemporal circadian dynamics inside this tiny nucleus can yield to circadian arrhythmia akin to cardiac arrhythmia, although its time scale is way too different. Thus, it will be important to know what are the essential components conferring their phase coherence, not to mention their entrainment ability?

The frequency-locking and phase-coherence of the SCN cell population are supposedly achieved by not just one but a few different cell-to-cell signaling mechanisms, and action potential (AP)-mediated connectivity is known to be critical. For example, silencing AP communication by administering Tetrodotoxin (TTX) or Pertussis Toxin (PTX) disintegrates large, coherent SCN waves into many, much smaller, turbulent wave segments and significantly impairs the overall phase coherence as we will discuss more shortly [7, 8, 9]. This seems natural, as the SCN clock cells are neurons, but quite intriguing because of the very disparate time scales related to the “fast” action potentials and the “slow” circadian rhythm. In principle, the AP-mediated connectivity can be long-ranged to provide a mean for the communications among very distant clock cells [10], thereby conferring the overall phase-coherence. Alternatively, structured clock cell network can undertake a more complex role: In a way, the SCN is an input-output device, which receives seasonal as well as geophysical day/night cycle information, processes them, and relays its output to various parts in the brain that in turn regulate appropriate circadian rhythms for the whole body; in doing so, the internal network structure may provide a mechanism for the location dependent phase dispersal, although this possibility is largely unexplored.

So far only a few experimental studies have addressed the morphology of SCN neural connectivity. One of them was a pioneering optogenetic mapping study by Fan *et al*. [11]: They reported that an SCN neuron would receive input from a given presynaptic VIP+ neuron with a probability of 0.06%; this was estimated by assuming an “unique binomial distribution.” Knowing the number of VIP+ neurons is approximately 1,100 within an SCN [12], we estimate that, on average, an SCN neuron receives only about 0.66 (= 0.06%×1,100) VIP+ presynaptic connections. In addition to the sparseness, Fan *et al*.’s analysis also suggested that afferent VIP+ connections are biased toward a subset of neurons. About the same time we also carried out an optogenetic connectome study of our own using a different experimental platform and protocol on slice cultures of neonatal rat SCNs to obtain two important statistics concerning the SCN network morphology [13]: the number of afferent connections and the distances to presynaptic clock cells projecting to a postsynaptic clock cell. With the statistics, we estimated the average indegree *k*^*in*^ per each clock cell to be 8.9, a number considerably larger than the estimated value of 0.66 based on the work of Fan *et al*. [11]. But our statistics included all different types (i.e., VIP- as well as VIP+) of connections within organotypic cultures of P3-5 rats, while Fan *et al*. [11] used acute brain slices of P25-35 mutant mice expressing ChR2 channels specifically either for VIP+ neurons or AVP+ neurons. As far as we know, these two reports are the only cases directly looking at the clock cell network connectivity within the SCN based on an optogenetic mapping technique. In fact, the optogenetic connectome study probing the SCN network connectivity is technically very challenging and extremely time demanding [13]. In addition, the mapping technique inherently cannot give any information about efferent connections (eg, outdegree *k*^*out*^).

Many years ago, Jacomy *et al*. [14] conducted elaborate analysis on a set of double immunocytochemistry images to report, on average, 5.4 VIP+ contacts on AVP+ cells and 1.7 AVP+ contacts on VIP+ cells for the SCNs of adult rats. These figures were based on presumed axosomatic contacts only; therefore, they could have been underestimated. On the contrary, they could have been overestimated as the small contacts identified in the immune-stained images do not guarantee a functional connection. More recently, Abel *et al*. [15] had a different approach for deciphering the SCN cell-cell network morphology: They inferred functional cell-cell connections in the SCN explants expressing bioluminescence reporter PER2::LUC, based on the so called ‘maximal information coefficient (MIC)’ scores. According to their analysis, the SCN network has “small-world network” characteristics and the hubs of this network are preferentially located in the (ventral/lateral) “core” region and cells in the outer (dorsal/medial) “shell” region are sparsely connected. The authors also claimed that the node degree distribution is exponential. The MIC connectivity analysis is based on the level of persistence of the phase difference as well as period relationship between two chosen cells: That is, the stronger the persistence is, the more likely they have a connection. It can be a false assumption, since a strongly persistent phase relationship between two distant cells does not guarantee a direct functional connection between them. In fact, as we will discuss shortly, the circadian SCN PER2::LUC phase waves, which repeat quite periodically in time generally have a very large correlation length. Besides, a persistent phase relationship between two cells can also be formed by a different cell-cell communication mechanism other than the AP-mediated connectivity.

So, there have been several significant efforts to decipher the cell-cell connectivity within the small nucleus, but we still have very little information about the coupling morphology within the SCN. Therefore, the goal of this paper is to use a mathematical model to develop the most realistic SCN network morphology, given the limited amount of information that has been accumulated. In order to achieve this goal, our investigation strategy is to 1) collect and combine previous experimental findings concerning the SCN, more explicitly, the statistics related to its known network structure and dynamic properties of individual cells, 2) re-evaluate the properties of SCN phase waves (published earlier by us and others) carefully, not only in their natural states but also after some extrinsic manipulations by drug treatments or temperature shock 3) design various mathematical model networks having a wide range of different network morphologies, and finally 4) sort out the most realistic model network(s) (among an extensive pool of all candidate networks) that support phase waves whose properties are good accord with those of the experimentally observed. Although it is not a completely new idea (see, for example, Ref. [16]), this work will be the first large-scale attempt to gain insights on the SCN cell-cell network leaning on its phase wave properties.

This paper is organized as follows. The next, second section discusses our new in-depth analysis on the properties of SCN circadian waves and some known properties of the SCN network morphology. The third section describes how we systematically build an extensive pool of 25,140 model SCN networks to be screened. The fourth section describes what are our network selection criteria and processes. The fifth section describes the properties of the selected model networks. Finally, in the last section we present the conclusion.

### Some known properties of the (rat and mouse) SCN

#### Circadian phase waves and TTX effect

**Fig 1:**
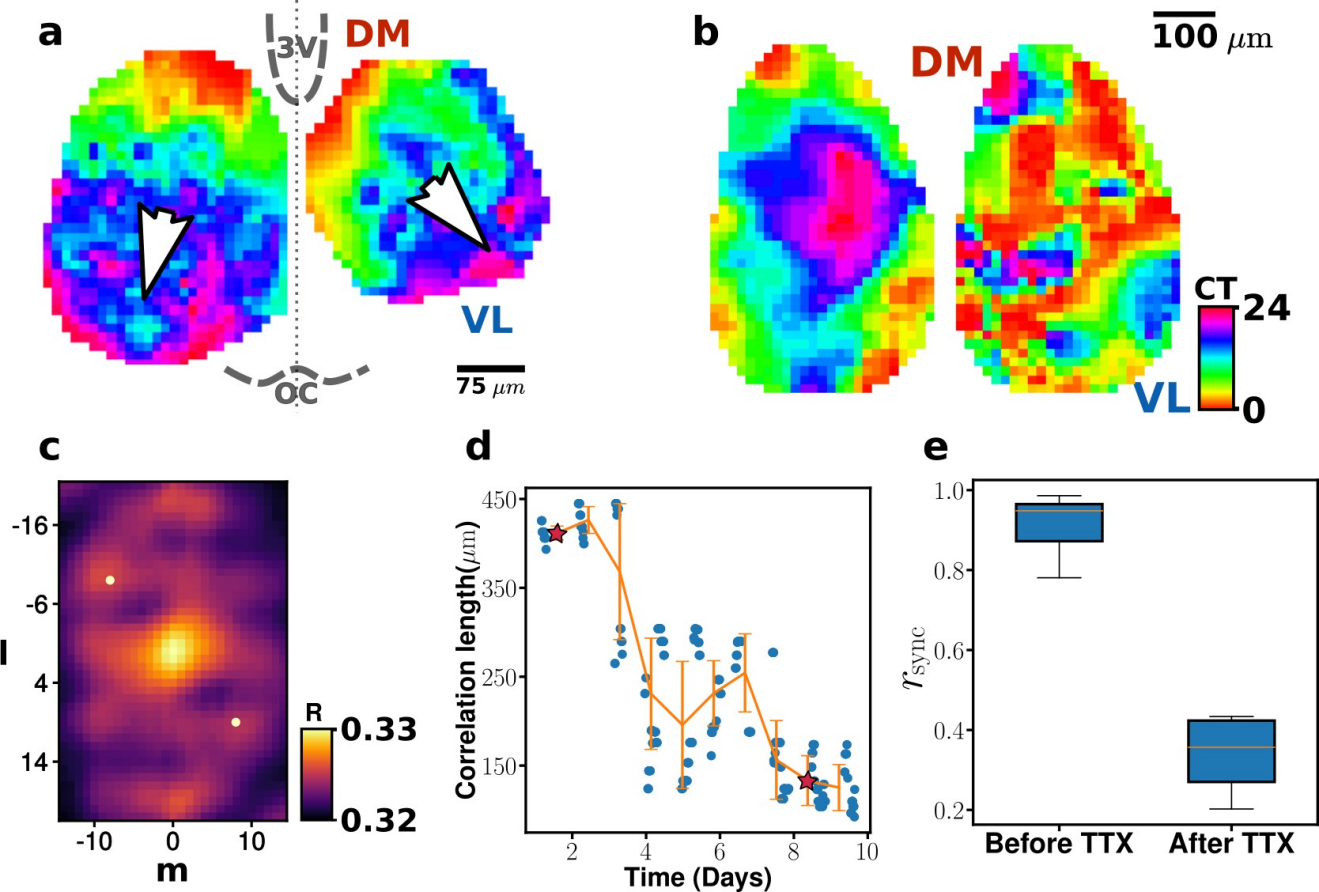
Circadian phase waves supported by mouse SCNs and their phase disintegration in the presence of TTX. (a) A 2D circadian phase map. The SCN circadian waves propagate typically from the shell (DM region) to the core (VL region) (see S1 Movie): The direction of each white arrowhead depicts the average instantaneous direction of phase wave propagation. To find the average direction, we compute velocity vector field (optical flow) from the set of reconstructed time-lapse images using a Gunnar-Farneback algorithm [20] and average the velocity vectors. (b) Two snapshot images of circadian phase taken at day 1 and day 8 of TTX application. (c) An exemplary 2D correlation map, corresponding to the phase map of Day 8 in (b)-right. The crosscorrelation $R(i,j)=\sum_{l=-(L-1)/2}^{\left(L-1\right)/2}\sum_{m=-(M-1)/2}^{\left(M-1\right)/2}I(l,m)\times I(l+i,m+j)$ is pseudo-color coded (*I* represents the image grey scale and *L* = 43 and *M* = 29 define length in pixels; one pixel = 14.4 *μm*). The two most prominent peaks around the center of the map are labelled by two white dots, and their average distance to the center is defined as 2D correlation length. (d) Time evolution of 2D correlation length over 10 days in the presence of TTX: The initial correlation length (~ 400 *μm*) gets significantly smaller (~ 140 *μm*) over several days. The star symbols mark the times for two phase maps shown in (b). The decay time constant *τ*_decay_ = 6.5 was obtained by fitting the data to an exponentially decaying function of $C_{0}e^{-t/\tau_{decay}}$. The solid orange lines connect average data points, each of which are binned over a 20-hour time window; error bars mark standard deviations (e) The synchrony measure $r_{sync}=\frac{1}{N}|\sum_{j=1}^{N}e^{i\phi_{j}}|$, where N is the total number of grid points, is much higher during unperturbed natural states (0.92±0.06) than TTX-treated steady states (0.42±0.23). The blue bands represent interquatile range, orange lines mark the median values (0.95 and 0.36, respectively), and black lines mark the full range of data. The 10 sample nuclei (of S1 Fig) are evaluated for the statistics. (The PER2::LUC signal data shared at https://github.com/johnabel/scn-resynchronization-data-2016 was used for (a) and (e), while the analysis presented in (b ~ d) are based on our (BJ and KJL) earlier experimental data published in Ref. [9]).

Fig 1A shows a snapshot image of PER2 luminescence level for a SCN coronal slice explant, which we have obtained by processing the experimental data published earlier by Abel *et al*. [15]. Briefly, the time series of (single cell resolution) PER2::LUC signals acquired at many different locations (i.e., cells) over a pair of nuclei (100 ~ 200 sites/nucleus) are processed to obtain the matching phase time series; simply, we set the times for two successive local maxima to be the bounds of a full phase range of [0, 2π] and assign a phase for each time point between the maxima as a linear function of time. Then, for each given time we construct a square grid map (grid mesh size = 10 *μm*) by linearly interpolating the signals in space. The same analysis was carried out also for 4 additional slice explants that were identically prepared (see S1 Fig)

The following two properties are noteworthy. First, for any given time the maps exhibit a significant degree of phase dispersal in space, often covering a complete 24-hour circadian range (see Figs 1A and S1). Second, all the slice explants seem to support a phase wave travelling, on average, from the dorsomedial (DM) region to the ventrolateral (VL) region [or from third ventricle (3V) to optic chiasm (OC)] as indicated by the white arrowheads in Fig 1A, which mark the average directions of phase wave propagation for the specific time instance of the map (see S1 Fig and S1 Movie). This phase wave property may not be universal but has been very commonly observed in many other previous studies employing a similar preparation [17, 18]. In fact, it has been suggested that there could be a pacemaking region in DM area of the SCN [19].

As an active approach towards understanding the mechanism underlying the SCN phase waves, one can perturb the system extrinsically and evaluate its consequence. Importantly, if the SCN gets treated with tetrodotoxin (TTX) which blocks the formation of action potentials (APs), thus, disrupting all AP-mediated couplings among the SCN clock cells, its phase coherence drops significantly (see Fig 1B for an example). For each phase map, a 2D correlation map is computed (see Fig 1C) from which a correlation length is measured. The decay of the correlation length of a TTX-treated SCN sample is plotted in Fig 1D, and its decay time constant is measured to be *τ*_*decay*_ = 6.5 (days). In other words, the disintegration of the initial phase wave takes several days; moreover, even for the (TTX-treated) long-term steady state, the 2D correlation length is still quite large; in fact, much larger (~ 150 *μm*) than that of a single SCN cell body (10 ~ 20 *μm*) (see Fig 1B, Day 8). The decrease in the correlation length caused by a TTX application is also evident if we compute the phase synchrony measure *r*_sync_ for all 5 samples (or 10 nuclei) of Abel *et al*. (see Fig 1E).

The analysis given in Fig 1B, 1E, and 1E clearly suggest that 1) AP-mediated clock cell-to-cell coupling is very significant as for keeping the phase coherence within the SCN and that 2) the SCN clock cell population should have additional short-range clock cell-cell coupling mechanism(s) in addition to the AP-mediated coupling. We believe that the additional mechanisms, which seems not hindered at all by the TTX application, is most likely mediated by the diffusion of some chemical substances: Several different candidates like VIP, AP5, NO or calcium signaling are already known for their relevance [21, 22]: For example, VIP receptor 2-null mutant mice clearly show spatial phase coherence [22]. However, as far as we know, the exact nature and effective diffusion constants of these relevant substances within the SCN have never been measured and reported.

Dynamic response of a given system being subject to an extrinsic physical perturbation could also provide us some useful information about the SCN. For example, if one delivers a global heat pulse shock (36.0 → 38.5°C increase, 6 hours) at an appropriate circadian phase, several ‘phase singularities’ can be generated (see S2A Fig). Phase singularities are an unusual spatial point around which the circadian phase covers a complete 24-hour cycle, and each of them serves as an organizing center of a spiral wave. They are special but quite a common feature in any spatially extended 2D systems of nonlinear oscillators or excitable media [23, 24]. Phase singularities observed in slice cultures of SCNs evolve spatiotemporally: After being created, they generally wander in space and ultimately disappear, typically by a pairwise collision or exiting off the sample boundary (see S2B Fig). Given a heat pulse shock, the number of phase singularities increases over 2 ~ 3 days beyond which it decays slowly over several days (see S2C Fig). As we will discuss later, we have performed a similar perturbation study for our *in silico* experiments to test if the result matches with that of the heat pulse experiment [9].

#### Overview on two known properties of the SCN clock cell network

Earlier, we (HK, CM, and KJL) carried out optogenetic connectome mapping analysis on slice cultures of rat SCN to characterize clock-cell network connections [13]. Briefly, organotypic SCN slice cultures were prepared using postnatal 3–5 days old Sprague-Dawley rats following a standard procedure as described in our previous paper [13]. After brain slices were cultured for about a week, ChR2 AVV virus (rAAV2/hsyn-hsyn-hchR2 (H134R)—EYFP-WPRE-PA) was injected for ChR2 transfection. The level of ChR2 expression reached its peak in about 2~3 weeks. Then, the ChR2 transfect culture samples were stimulated by a blue power LED light in a repetitive box-scanning modes via a digital micro-mirror array, while a target cell was whole-cell patched. The postsynaptic currents of the target cell, which were brought by light-induced action potentials of presynaptic neurons, were measured as the stimulating light scanned the entire culture systematically. The size and shape of the *receptive field* of each neuron were quite diverse. Figuring out the number of presynaptic neurons and their locations for a given receptive field, was not an obvious job to do and we used a clustering algorithm to segment the receptive field to obtain the number of independent sources (i.e., presynaptic neurons): A hybrid measure D, which combines the physical distance and the difference in the current amplitudes of two different grid sites, was defined and used for the clustering process.

**Fig 2:**
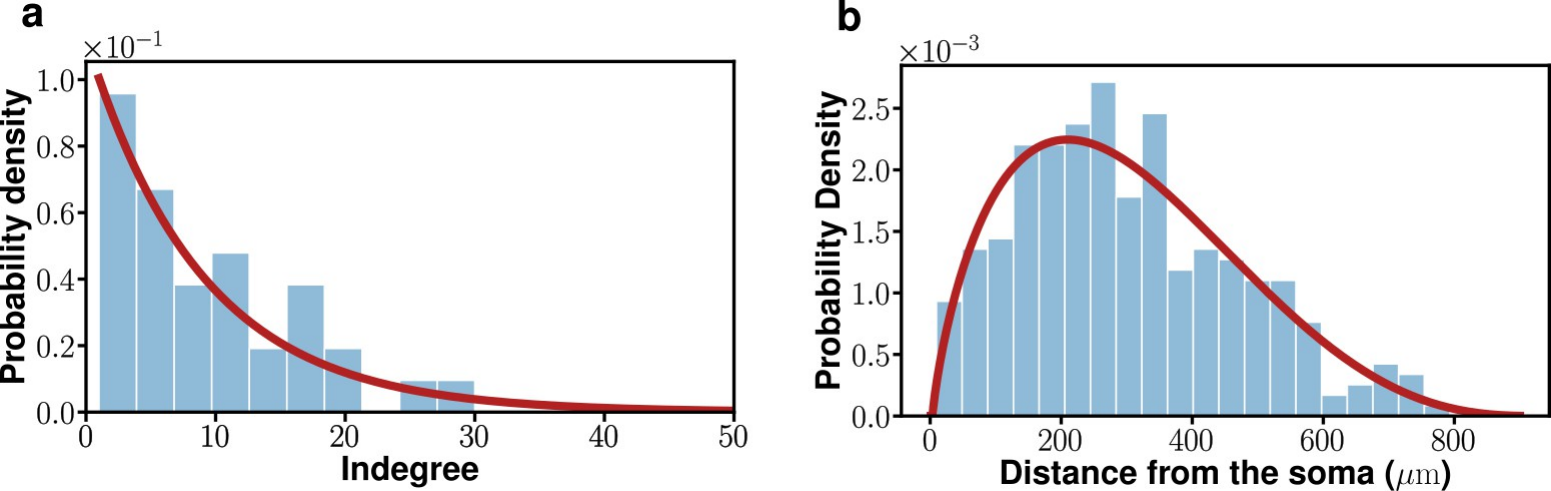
Indegree *k*^*in*^ probability density function (*pdf*) (a) and *pdf* of the distances to pre-synaptic neurons (b) of neonatal SCN clock cells. The solid red line in (a) is a fit to an exponential pdf (with a mean of 8.9). The distance in (b) refers to the relative Euclidean distance from a postsynaptic neuron to its presynaptic neuron(s) (solid red line: a fit to beta *pdf*; the median is ~205 *μm*). (This figure is adapted from Ref. [13]).

Two key findings were: First, the indegrees of postsynaptic cells followed an exponential distribution with a mean value of 8.9 (see Fig 2A); second, on average, the presynaptic connection number density around a postsynaptic cell peaked around 200 *μm* and then fell off as a decreasing function of separation distance (see Fig 2B). Indeed, the space constant (~210.7 *μm*) of the number density was quite large, and long-range (> 210.7 *μm*) neural connections were quite common (> 66.9%) within the SCN. Of these two statistics, the indegree distribution will be built into all the potential model networks we will consider, while the distribution of the distances to presynaptic cells will be used as a validation for successful model networks. Here we should point out that these statistics were obtained with rat SCNs while the phase waves discussed with Fig 1 were observed with mouse SCNs. But for the level of details associated with the mathematical model we are to develope, we ignore potential differences between rat and mouse SCNs.

### Building an archive of potential SCN clock cell network models

#### Determining the size of clock cell population and individual oscillator type

Confronting daunting challenges and limitations in acquiring the SCN connectome, we have decided to explore a large set of model clock cell network morphologies, in a systematic way, and find out which are the most appropriate candidates that would faithfully reproduce the experimentally observed phase wave properties discussed in Fig 1 (and S1 and S2 Figs). First, we need to set the system size, in other words, the number of clock cells within the model SCN properly. As for the organotypic slice cultures of the (3~5 neonatal rat) SCN that were used for the connectome statistics discussed in Fig 2, we estimate that one SCN nucleus of a slice culture is composed of approximately 5,300 neurons (this figure is based on the measured 2D surface area of 3.5×10^5^ *μm*^2^ and thickness of 1.2×10^2^ *μm* of one sample nucleus) and an approximate size of single clock cell of 8×10^2^ *μm*^2^. Accordingly, we have assumed that our model nucleus is comprised of 5,000 clock cells and their (square) grid distance is 8.45 *μm*. These are crude estimates, but we find that even 20% changes of these values do not alter our conclusion in any significant way (see S1 Text). Second, we have set the individual SCN clock cells to be a nonlinear Poincaré oscillator that is described by the following set of equations:

$$\begin{array}{c} \frac{dx_{i}}{dt}=\gamma (\mu_{i}-r_{i})x_{i}-\omega_{i}y_{i}\\ \frac{dy_{i}}{dt}=\gamma (\mu_{i}-r_{i})y_{i}+\omega_{i}x_{i}, \end{array}$$ (1)

where $r_{i}=\sqrt{x_{i}^{2}+y_{i}^{2}}$, *μ*_*i*_ is the Hopf bifurcation parameter, *γ* controls the rate of relaxation to the stable oscillation, and *ω*_*i*_ controls the period of each individual oscillator. We have assumed that *μ*_*i*_ is distributed over the entire population according to a normal distribution *N*(0.30, 0.54) so that 70% of the population are self-sustained Hopf oscillators while the remaining 30% are damped oscillators which can be entrained by the autonomous oscillators. This proportionality reflects the experimental data reported by Hirata *et al*. [5]. Earlier, Welsch *et al*. [4] measured the circadian periods of dissociated SCN clocks cells to have a wide range of 22.7 ~ 29.2 hours. Accordingly, we have assumed *ω*_*i*_ to follow a normal distribution of *N*(24, 2) over the whole population. We have not considered using a more realistic SCN clock-cell oscillator model such as Becker-Weimann model [25] or Kim-Forger model [26], since we believed that the simple, generic 2-variable Poincaré oscillator would be sufficient for achieving the level of details that we were going to characterize. In fact, similar Poincaré oscillator models have been used extensively providing pertinent insight on circadian properties, for example, see [27].

#### At least two different clock cell-cell communication mechanisms

We point out again that even when a TTX application blocks AP-mediated synaptic coupling, the SCN phase wave’s correlation length is still very large (~ 140 *μm*) (see Fig 1D). Therefore, there must be some alternative cell-cell communication mechanisms conferring the local phase coherence, and we believe that it could be mediated by non-synaptic diffusion neurotransmission (NDN) [28], cell-cell communication mediated by the diffusion of some neurotransmitters (and/or neuroactive substances) in the extracellular medium leading to relevant, extrasynaptic, receptors. For the SCN, there are several good candidates for the NDN cell-cell coupling: There has been considerable evidence that VIP [29], AVP [30], GABA/glutamate [31, 32, 33], and free calcium [34] serve an important role in the SCN clock cell-cell signaling, although how these neurotransmitters mediate the coupling is still largely unknown [35]. Nitric oxide (NO) is also a well-known substance for coupling among clock cell networks with its rapid transcellular effects [36].

Therefore, it is very conceivable that the SCN supports at least two different cell-to-cell coupling mechanisms: local diffusive coupling among adjacent neighboring cells and potentially very far-reaching, extensive, AP-mediated network coupling. In fact, a similar viewpoint was taken previously in other SCN modelling studies [16, 37]. Thus, we assume the following set of two equations can describe the network dynamics:

$$\begin{array}{c} \frac{dx_{i}}{dt}=\gamma (\mu_{i}-r_{i})x_{i}-\omega_{i}y_{i}+D\nabla^{2}x_{i}+K\sum_{j=1}^{N}A_{ij}x_{j}+Lsin(\Omega t+\phi )\\ \frac{dy_{i}}{dt}=\gamma (\mu_{i}-r_{i})y_{i}+\omega_{i}x_{i}+D\nabla^{2}y_{i},\;\mathrm{where}\;r_{i}=\sqrt{x_{i}^{2}+y_{i}^{2}}. \end{array}$$ (2)

The terms that start with *D* represent a diffusive coupling, while the term beginning with *K* is for the AP-mediated synaptic coupling, which can be very far reaching. *A*_*ij*_ represents an adjacency matrix with its elements either 1 (connection) or 0 (no connection). A common cell-cell coupling strength is set by a parameter *K*: that is, we consider only uniformly weighted networks. For our simulations, the Laplacian operator *∇*^2^ was approximated with a standard 4-neighborhood finite difference scheme on a two-dimensional square grid. Diffusion coefficient of some neurotransmitters like glutamate is well characterized, while those for large peptides like VIP are less known: One earlier report estimates *D*_VIP_ ~ 0.25 μm^2^/ms and *D*_GABA_ ~ 0.9 μm^2^/ms within extracellular fluid [38, 39]. The diffusion coefficients of the neurotransmitters would affect the strength *D* of the diffusive coupling of circadian state variables (e.g., the concentration of Per2 clock genes), but *D*_VIP_ or *D*_GABA_ themselves are not the same as the effective *D*, which can depend on many other cell-intrinsic factors [40, 41, 42]. In this study, we can indirectly estimate a reasonable value for *D*, based on the measured spatial correlation length of the turbulent phase waves of the TTX-treated SCNs, and compare it with that of model simulation; as for the model system, we simply set *K* = 0 to mimic the TTX-treated situation. On the other hand, there is no direct way to determine the strength *K* of the AP-mediated cell-to-cell coupling. In fact, the great challenge ahead of us is to estimate the strength *K* as well as the morphology of the AP-mediated SCN network to recapitulate the real SCN circadian phase waves.

Finally, the last term Lsin *Lsin*(*Ωt*+*ϕ*) in the first equation of (Eq 2) represents a common external forcing given to all cells in the core region, and this term will be included only when we want to evaluate how quickly the model SCN network readjusts to reestablish its overall core/shell phase pattern. That is, this term mimics an extrinsic circadian forcing such as the geophysical light-dark cycle. *L* and *Ω* are, respectively, the strength and angular frequency of the forcing, and *ϕ* is the relative circadian with respect to the autonomous circadian rhythm.

#### Systematic build-up of an extensive library of potential SCN clock cell network morphology

As for the AP-mediated synaptic coupling, all we have acquired so far are the indegree *pdf* and that of the distances to presynaptic neurons (Fig 2). But these two are not at all sufficient for defining the SCN network morphology. For instance, we do not know what is the outdegree (*k*^*out*^) *pdf* like. Nothing is known about various graph-theoretical measures, such as assortativity or clustering coefficient, for the real SCN clock cell networks. So, the best strategy for unraveling the actual SCN network morphology via mathematical modeling could be 1) an exhaustive scan of parameters for the realization of various network morphologies, 2) run computer simulations with the coupled oscillator model equation (Eq 2), and 3) find the cases that generate phase waves whose properties closely match those of real SCN phase waves observed in experiments. Key SCN network properties under examination include degree of circadian phase synchrony; overall direction of phase wave propagation; phase resetting property of the coupled oscillators to an external periodic forcing [mimicking a subject being forced by a sudden shift in geophysical circadian time (as in a jetlag)]; and the decay rate of the number of phase singularities created by a global perturbation.

**Fig 3:**
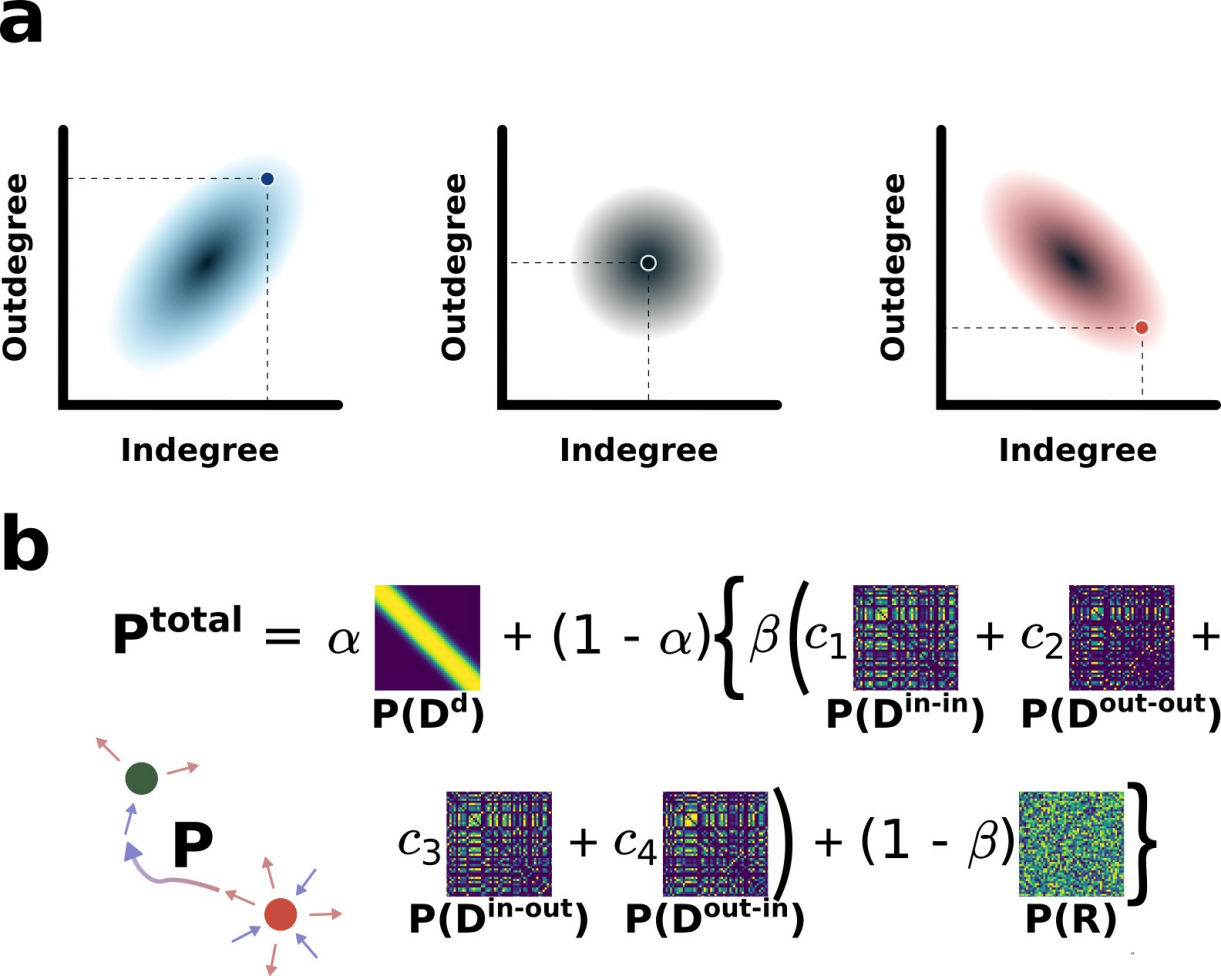
Generating a comprehensive archive of model SCN clock cell-cell networks (schematic illustration). (a) Three different (positive, random, negative) types of *k*^*in*^−*k*^*ou*^ correlation. (b) The total connection probability matrix *P* is a linear combination of *P*(*D*^*d*^), *P*(*D*^in-in^), *P*(*D*^out-out^), *P*(*D*^in-out^), *P*(*D*^out-in^), which maximizes the clustering coefficient, *k*in–*k*^in^, *k*out—*k*^out^, *k*in—*k*^out^, and *k*out—*k*^in^ assortativity coefficients, respectively, and *P*(*R*) which is a random connection probability matrix. *α*, *β*, *c*_1_, *c*_2_, *c*_3_ and *c*_4_ are weighting parameters controlling the relative significance of the involved terms.

Fig 3 schematically illustrates how we have built a comprehensive archive of model networks in a systematic way. First, we have categorized potential candidate networks primarily into three different classes based on different (positive, random, or negative) *k*^*in*^−*k*^*out*^ correlations as schematically illustrated in Fig 3A: This is because degree correlations can be the most fundamental property of a complex network after the degree distribution function itself [43]. (As we will discuss later, *k*^*in*^−*k*^*out*^ correlation has a critical effect on the level of phase coherence of the model SCN). So, for example, when the degree correlation is positive (negative), the larger (smaller) *k*^*in*^ a node has, the larger (smaller) *k*^*out*^ it will have. Since the indegrees of the SCN clock cells in the earlier experiment were found to follow an exponential distribution with an average value of 8.9 [13], we randomly assign an indegree for each of all 5,000 model cells to follow the same exponential distribution. For generating our archive of model SCN networks, we have considered 5 (3) different positive (negative) *k*^*in*^−*k*^*out*^ correlation levels and 1 randomly correlated case, which are labeled by pos(10), pos(20), pos(30), pos(40), pos(50), neg(10), neg(20, neg(30), and none(0), respectively (Sec. 1 in S1 Text describes how we systematically generate these different cases).

Once both *k*^in^ and *k*^out^ are determined for every node, we are to determine which node connects to which nodes–the main process that will finalize all network features. In doing so, we allow neither “self-looping” of a node nor “bidirectional connection” between two nodes, as they are known to be rare [44]. Then, we have considered the local graph-theoretical measures, clustering coefficient and assortativity coefficient, to be the two most important features characterizing a complex network besides the type of degree correlation. Clustering coefficient *C* of a node quantifies how close its neighbors are to being a complete graph–a tendency to cluster together (see Methods for its mathematical definition). For a given population of nodes, whose *k*^in^ and *k*^out^ are predetermined, we can systematically obtain a connection probability matrix *P*(*D*^*d*^) with which one can generate a network having the maximum value of clustering coefficient (Sec. 2 in S1 Text).

Assortativity is another important measure for a given node to connect to others having a similar degree (see Methods for its mathematical definition). We can define four different degree-based (*k*in–*k*^in^, *k*out—*k*^out^, *k*in—*k*^out^, and *k*out—*k*^in^) assortativities: *r*^in-in^, *r*^out-out^, *r*^in-out^, *r*^out-in^, respectively. Basically, following a similar procedure, which was used for obtaining *P*(*D*^*d*^), we can systematically obtain a connection probability matrix *P*(*D*^in-in^), maximizing *k*in–*k*^in^ assortativity (second thumbnail in Fig 3B; see S1 Text for further detail). Likewise, we can obtain the other edge connection probability matrices, *P*(*D*^out-out^), *P*(*D*^in-out^), and *P*(*D*^out-in^), which would maximize *k*out—*k*^out^, *k*in—*k*^out^, and *k*out—*k*^in^ assortativity, respectively (Sec. 3 in S1 Text). We have also considered some randomness in the edge connections by introducing a random connection probability matrix *P*(*R*). Fig 3B shows an exemplary thumbnail set of all 6 different probability matrices.

Finally, we linearly combine them together to define a ‘total edge connection probability matrix’ as illustrated in Fig 3B:

$P^{\mathrm{total}}=\alpha P(D^{d})+(1‐\alpha )\beta \{c_{1}P(D^{\mathrm{in}‐\mathrm{in}})+c_{2}P(D^{\mathrm{out}‐\mathrm{out}})+c_{3}P(D^{\mathrm{in}‐\mathrm{out}})+c_{4}P(D^{\mathrm{out}‐\mathrm{in}})+(1‐\beta )P(R)\},$ where *α* ∈ [0, 1], *β* ∈ [0, 1], and *c*_1_, *c*_2_, *c*_3_, and *c*_4_ (*c*_1_+*c*_2_+*c*_3_+*c*_4_ = 1) are a set of 6 parameters that weigh the relative significance of different graph-theoretical measures and randomness for the trial network. If we set *α* to be a large (small) value near 1 (0), the network will be optimized for the strongest clustering coefficient (assortativity). On the other hand, *β* controls the level of random connectivity over network assortativity: The larger *β* is, the more significant the assortativity will become for the network. Then, *c*_1_, *c*_2_, *c*_3_, and *c*_4_, weigh the relative significance of different types of assortativity. Finally, we build efferent edge connections $k_{i}^{\mathrm{out}}$ with *P*^total^ (for a given set of 6 parameter values) in a random sequence of all nodes until all efferent, as well as afferent edge connections, become consistent with the preassigned set of all $\left(k_{i}^{\mathrm{in}},k_{i}^{\mathrm{out}}\right)$. For our current investigation, we have considered 5,376 (= 32×168) different node-node connection matrices with 32 different sets of equally spaced (in log-scale) levels for *α* and *β* and 168 different sets of {*c*_*i*_}.

#### Location assignment within a 2D model nucleus and number of intra and inter-subdivisional edges

**Fig 4:**
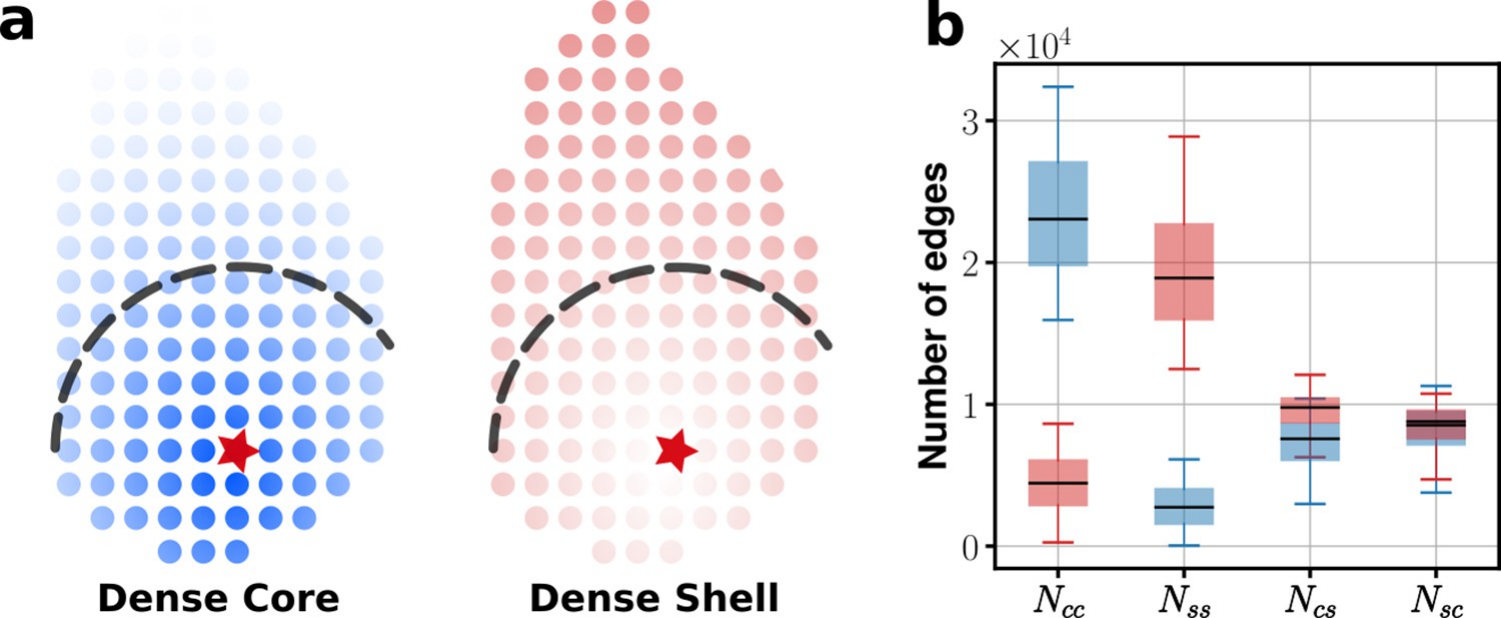
**Schematic illustration of two different node configurations in (a) and the number of efferent connections within two compartments [core-to-core (***N*_*cc*_**) and shell-to-shell (***N*_*ss*_**)] and those across them [core-to-shell (***N*_*cs*_**) and shell-to-core (***N*_*sc*_**)].** The red stars in (a) mark the central position (*p*^*c*^, *q*^*c*^) of the SCN. The blue (red) color represents the case of dense core (shell) and the different levels of shade represent the corresponding values of degree. The data shown in (b) is for the cases when *k*^in^ is positively correlated with *k*^out^ (blue: core dense; red: shell dense). The subdivision ‘core’ is the area occupied by all the nodes that are within 245 *μm* from (*p*^*c*^, *q*^*c*^); the boundary between two subdivisions is marked by a dashed line. The color shades schematically illustrate the size of *k*^total^ (see S1 Text for further detail).

Once we have established a node-node connection matrix, we need to assign a specific location for each one of the nodes inside our model SCN nucleus. The inference made by Abel *et al*. [15] suggests that the clock cells in the core region have a higher degree than that of the cells in the shell region. As far as we know, however, there is no direct experimental confirmation about it, and here we have explored the following two different possibilities: 1) The SCN clock cells in the core region have a higher degree than that of the shell region or 2) the opposite case. Again, our model SCN is a 2D square-grid mesh shaped in the actual coronal section of a rat SCN slice culture [13], as illustrated in Fig 4A. We can systematically arrange the distance of a node from the center (star marks in Fig 4A) according to its value of *k*^*total*^, *k*^*in*^ or *k*^*out*^ (see S1 Text). All together, we have considered 30 different ways of assigning nodes to the grid space of the model SCN, incorporating 5 different levels of randomness.

Although rat (or mouse) SCNs are composed of several different subregions, often they are viewed just as a sum of two distinctive subdivisions, the core and shell [12, 16, 45]. Accordingly, we divided the area of our SCN model almost equally into core and shell areas and defined the center point of the core area as (*p*^*c*^, *q*^*c*^): 55% (45%) of the total number (5,000) of nodes of our model SCN belongs to the core (shell) (see Fig 4A). Once we place all nodes within the nucleus, we wonder how many efferent (or afferent) connections are formed between the core and the shell or within each subdivision, an important property of the SCN which has not been explored much through experiments, for the various models of our network archive. Shown in Fig 4B are the results based on the 5 model networks in which *k*^in^ is positively correlated with *k*^out^ (i.e., one sample from each of pos50, 40, 30, 20, 10 cases). For the case of ‘Dense Core’ (colored blue), the nodes in the core are designed to have a larger *k*^in^ as well as a larger *k*^out^ than what the nodes in the shell would have: Consequently, there will be many more efferent connections projecting from the core to the same core (*N*_*cc*_) compared to those projecting from the shell to the same shell (*N*_*ss*_); and the number of edges reaching the shell from the core *N*_*cs*_ or that reaching the core from the shell *N*_*sc*_ would be smaller than *N*_*cc*_ but larger than *N*_*ss*_. For the case of ‘Dense Shell,’ on average, *N*_*ss*_ > *N*_*cs*_ (≈*N*_*sc*_) > *N*_*cc*_ (see red bars in Fig 4B), as one would expect. When *k*^in^ is negatively correlated with *k*^out^, the results are quite complex: 1) for different types of network, both *N*_*cs*_ and *N*_*sc*_ vary widely while *N*_*cc*_ and *N*_*ss*_ do not; 2) *N*_*cs*_ ≫ *N*_*sc*_ (when *k*^out^ ≫ *k*^in^ for the core); and similarly, 3) *N*_*sc*_ ≫ *N*_*cs*_ (when *k*^out^ ≪ *k*^in^ for the core) (not shown).

#### Too many network morphologies to be considered: uniform sampling issue

All together, we have 9 different *k*in—*k*^out^ relations; 5,376 sets of 6 weighting factors for the connection probability matrix; and 30 different ways to assign the nodes to their physical locations. Taken together, we have 1,451,520 different model SCNs to simulate. This is too many to be simulated with our limited resources. Moreover, scanning the six, *α*, *β*, *c*_1_, *c*_2_, *c*_3_, and *c*_4_, uniformly does not yield a uniform distribution in the 5-dimensional space of graph-theoretical measures (*C*, *r*^in-in^, *r*^out-out^, *r*^in-out^, *r*^out-in^). So, after computing all 5 graph-theoretical measures for all candidate networks, we uniformly under-sample the candidate networks in the 5D metric space of the measures (i.e., one sample per one 5D grid mesh) to reduce the total pool of candidates down to 25,140. This under-sampling process assumes that two different networks that are close in the 5-dim space of (*C*, *r*^in-in^, *r*^out-out^, *r*^in-out^, *r*^out-in^) would produce a similar spatiotemporal circadian phase dynamics, and we have confirmed that indeed they do (see S1 Text for further detail).

**Table 1:** The number of different SCN network morphologies that are selected for the SCN model simulation and screened for their suitability as an SCN model [listed for different levels of indegree-outdegree correlation; see Fig 3A (and Sec. 1 in S1 Text) for the meaning of the labels].

*k*^in^—*k*^out^ correlation	neg30	neg20	neg10	none	pos10	pos20	pos30	pos40	pos50
**Number of cases (total: 25,140)**	2,340	2,520	2,160	4,980	5,040	3,840	1,620	1,200	1,440

#### Determination of the model parameters *γ*, *D*, and *K*

Our mathematical model SCN (Eq 2) includes three scalar parameters *γ*, *D*, and *K*, which are the Hopf bifurcation parameter, effective diffusion coupling strength of (messenger molecules), synapse-mediated cell-to-cell coupling strength, respectively. We will estimate the value of *γ* and *D* using the information about the average 2D phase correlation length ~ 140 *μm* of the TTX-treated SCN PER2::LUC waves (see Fig 1D). But, at this point of time we should indicate that the estimation of ~ 140 *μm* was based on slice cultures of mouse SCNs which are smaller than those of the rat SCNs used for the SCN statistics given in Fig 2. Since we are to model rat SCNs, we project the average correlation length of the phase waves within the TTX-treated rat SCNs to be a larger value of 210 (= 1.5×140) *m*, for which we have simply assumed that circadian phase waves just rescale in proportion to the square root of the 2D-projected SCN area: The average area (360,000 *μm*^2^) of the rat SCNs used for Fig 2 is about 2.2 times larger than that (164,000 *μm*^2^) of mouse SCN used for Fig 1D.

**Fig 5:**
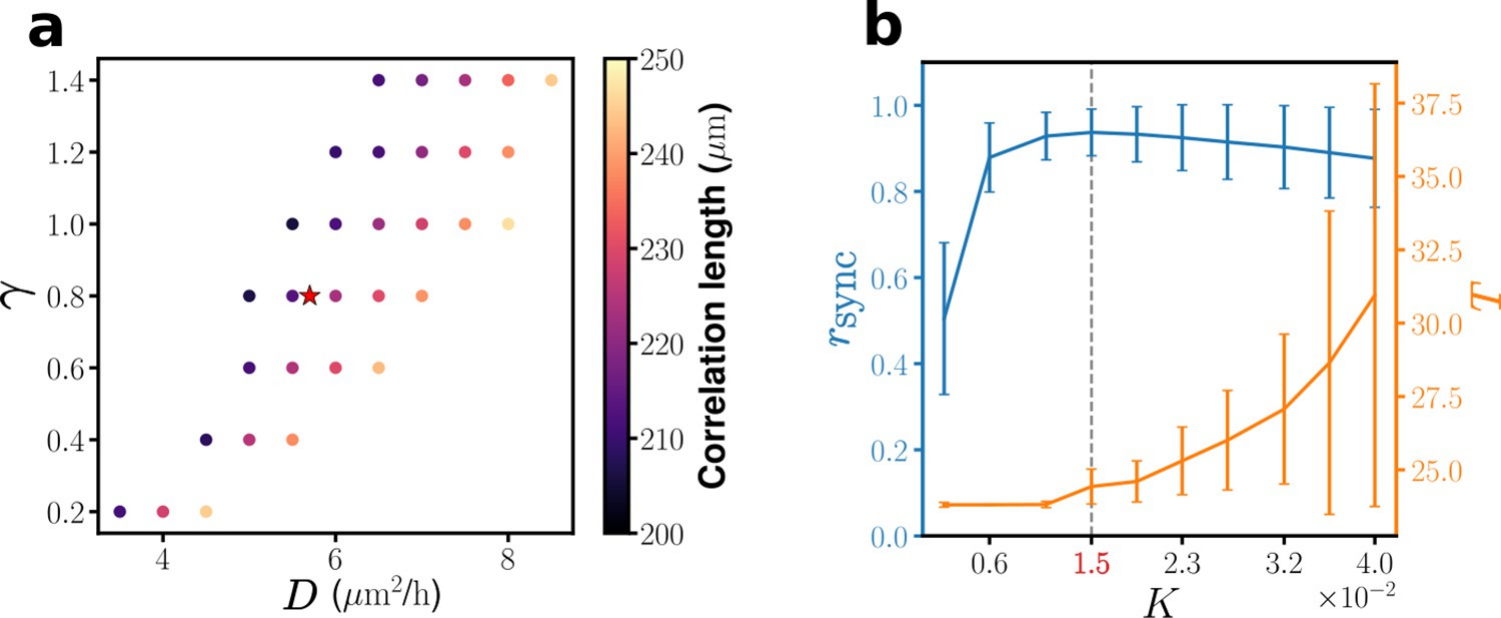
Determination of three key parameters *γ*, *D*, and *K* guided by the analysis of experimental data. (a) Colormap of the mean correlation length of diffusion-only mediated phase waves, in the absence of network coupling (i.e., *K* = 0). The star symbol marks the position of the selected choice of (*γ*, *D*) = (0.8, 5.7 μm^2^/h), which be used for all full (i.e., *K* ≠ 0) model simulations. (b) The degree of (population) phase synchrony *r*_sync_ and (population) mean period *T* of the model SCN. The error bars represent the standard deviations of the values covered by all candidate model networks listed in Table 1. The dashed line in (b) marks the value of *K* = 1.5×10^−2^, with which we could recapitulate several key properties of experimentally observed circadian phase waves.

We set *K* = 0 in the model SCN simulations, mimicking the blocking of AP-mediated coupling by the application of TTX to SCN samples; run just diffusion-coupled networks of oscillators for various sets of (*γ*, *D*) to obtain corresponding circadian phase waves and calculate their average phase coherence lengths as given in Fig 5A. Then, we select a pair of (*γ*, *D*) = (0.8, 5.7 *μm*^2^/*h*) that results in an average phase coherence length of 214 *μm*, a value close to our crudely, yet empirically estimated value of 210 *μm*. Of course, the set of (*γ*, *D*) could be chosen differently given only one constraint; that is, an additional constraint which is independent from the constraint of phase coherence length would be needed for defining a unique set of (*γ*, *D*). *γ* determines how quickly the synchronized oscillation of individual nodes returns to their intrinsic limit cycle after being disconnected. We have picked one of the *γ* where synchrony drops to ~ 0.6 after ten days of disconnection. Then, we have selected a value of *D* that gives the correlation length of 214 *μm* corresponding to the selected value of *γ = 0*.*8*.

A similar strategy was used for determining the value *K*. We have run the SCN model simulation many times by varying the value of *K* systematically from 2.1×10^−3^ to 4.0×10^−2^ in a step size of Δ*K* = 4.2×10^−3^ and measured the degree of overall phase synchrony *r*_sync_ and mean period *T* as shown in Fig 5B. The standard deviation *ΔT* as well as *T* itself increases monotonically as a function of *K*. On the other hand, *r*_sync_ is a nonmonotonic function of *K*, dramatically increasing up to *K*~1.5×10^−2^ and decays very slowly thereafter. Considering that the SCN would normally support 1) 24.0 < *T* < 25.5 h and 2) *r*_sync_ > 0.9 (see Fig 1E) [16], we have determined the suitable *K* range to be 1.0×10^−2^~2.0×10^−2^. We further pin down the most suitable *K* value to be *K* = 1.5×10^−2^ by checking the decay time constants of the number of (perturbation-induced) phase singularities: Among all cases, the choice of *K* = 1.5×10^−2^ maximizes the number of model networks 1) that have the recovery time constant falling within 2~3 days, the range that we have confirmed through the analysis on our previous perturbation experiments and 2) that do support the required criteria on the period as well as the level of phase synchrony (further details are discussed later with the condition 1.5 **<** *τ*_ps_ **<** 5.0 days).

## Results

Up to now, basically we have 1) built a comprehensive archive of model networks, incorporating what we know so far about the (neonatal, 3~5 days) rat SCN clock cell network (Fig 2A), 2) analyzed some basic graph theoretical features of the diverse network morphologies of the network archive, and 3) determined the values of three key parameters of the model (Eq 2). From now on, we are to determine which network morphologies would be appropriate for supporting the experimentally confirmed phase wave properties. Note that the experimental result shown in Fig 2B is saved for the validation of the selected group of networks at the end.

### Imposition of two conditions: the shell’s phase is ahead of the core’s phase (*Δt* > 1 hr) and the core recovers more quickly than the shell (*Δτ*_rec_ > 0)

As we have fixed the parameters *γ*, *D* and *K*, we are now ready to undertake a massive set of numerical computations with various network morphologies, covering 9 different types of indegree-outdegree relations and different levels of clustering and assortativity coefficients, as listed in Table 1. All simulations were carried out in a personal PC (CPU: AMD Ryzen 9 3900X, GPU: GeForce RTX 3080) based on home-built codes written in Julia (and) Python.

**Fig 6:**
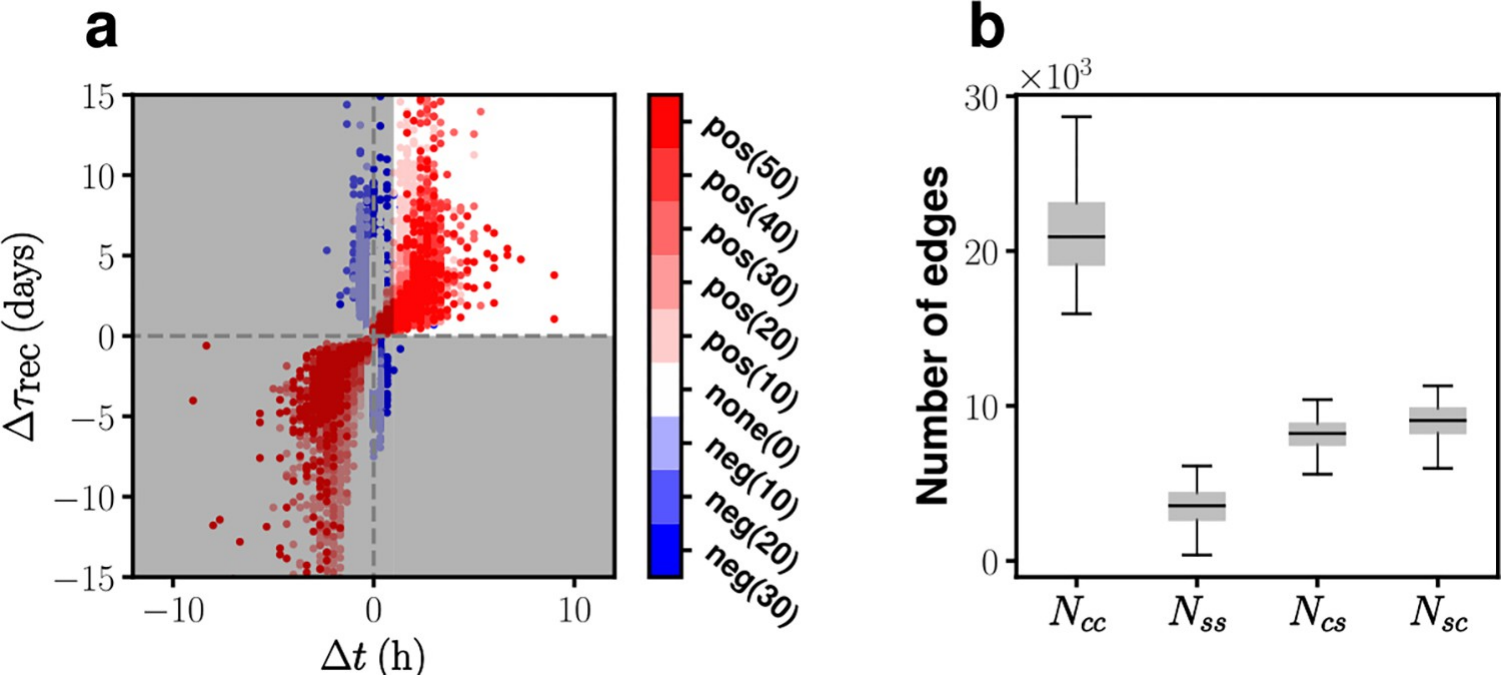
Filtering the pool of network morphologies by imposing constraints on *Δt* and *Δτ*_rec_. (a) point locations in the space of *Δt* and *Δτ*_rec_, each of which corresponds to a network model listed in Table 1. Different types and levels of indegree-outdegree correlation are color-coded with blue and red. Network models falling into the gray-shaded region were excluded. (b) Numbers of intra-subdivision (*N*_*cc*_ and *N*_*ss*_) and inter-subdivision (*N*_*cs*_ and *N*_*sc*_) edges for the group of selected model networks satisfying *Δt* > 1 hr and *Δτ*_rec_ > 0. (the number of points in the highlighted white area = 1,084; total number of points = 25,140).

All simulations begin with uniformly distributed, but randomly scattered, circadian phases over the entire system and we have confirmed that the steady state behavior of the system is not sensitive on different (random) initial conditions (see Fig D in S1 Text). Once the numerical integration reaches to a steady state, we determine if the model network morphology is suitable or not by evaluating the properties of the resulting phase wave. We first quantify the overall phase difference *Δt*, between the core and the shell: *Δt* is defined to be the time *τ*^max^ that maximizes the auto-correlation function ∫*Φ*_core_(*t*)*Φ*_shell_(*t*−*τ*)*dt*, where *Φ*_core_(*t*) and *Φ*_shell_(*t*) represent the mean population phase of the core and the shell, respectively. We require that the shell’s phase is ahead of the core’s phase at least 1 hour (*Δt* > 1 hr) for a valid model network: Again, the lower bound 1 hr is chosen according to our comprehensive survey on the SCN circadian phase waves [16, 18]. Note that this is an additional requirement in addition to *r*_sync_ > 0.9 and 24.0 ≤ *T* ≤ 25.5 hr that we already imposed while fixing the parameter value of *K* = 1.5×10^−2^. Second, we evaluate how fast the subdivisions recover after the regional cell-to-cell phase coherence is disrupted with an application of TTX (and a subsequent wash). That is, we compute the recovery time constants $\tau_{\mathrm{rec}}^{\mathrm{core}}\;(\tau_{\mathrm{rec}}^{\mathrm{shell}})$ for *Φ*_core_ (*Φ*_shell_) and select those network morphologies satisfying ${\Delta \tau }_{\mathrm{rec}}=\tau_{\mathrm{rec}}^{\mathrm{shell}}{-\tau }_{\mathrm{rec}}^{\mathrm{core}}>0$; this requirement is also based on a general consensus that the core (as versus to the shell) recovers its phase coherence faster [15, 16]. Fig 6A plots all sets of (*Δt*, *Δτ*_rec_) calculated for all the network morphologies being considered in Table 1 and simulated. With the two requirements, *Δt* > 1 hr and *Δτ*_rec_ > 0 (the subregion with white background in Fig 6A), we have reduced the archive of network morphologies significantly from 25,140 down to 1,084. Interestingly, those chosen network morphologies have the following two properties: 1) indegree and outdegree are positively correlated; 2) the nodes in the core have many more edges than those in the shell (see Fig 6B, this issue will be discussed later in full detail).

#### Imposition of two additional conditions: the entrainment time to an external forcing (*τ*_ent_ < 10 days) and the decay time constant of phase singularities 1.5 < *τ*_ps_ < 5.0 days)

We require our mathematical model SCN to satisfy two additional properties. The first one is about ‘phase recovery ability,’ which is how quickly the model SCN can reset its intrinsic circadian phase to the phase of extrinsic oscillator forcing the SCN. This issue mimics the question of how quickly the biological master clock SCN can overcome a jetlag caused by a sudden shift in the phase of light/dark cycle. As for the phase resetting ability, we ask a realistic model SCN network to have the following property: Upon receiving a sudden time shift brought by an external sinusoidal forcing *Lsin*(*Ωt*+*ϕ*) with *ϕ* = 6 hr, *L* = 0.05, *Ω* = 2*π*/24, which is added only to the nodes in the core region through the variable *x*_*i*_ (see Eq 2), the cells in the core and shell initially desynchronize but gradually recover their original phase coherence and relative phase difference (not shown). *Ω*, *ϕ* and *L* are important parameters whose values are critical for the phenomenon of phase entrainment [46]. The choice of *ϕ* = 6 hr was in accordance with the relevant earlier experiment by Jeong et al. [9]; likewise, *L* = 0.05 was chosen to mimic the strength of the temperature perturbation used in the same experiment. Then, we require the phase coherence recovery time *τ*_*ent*_ of our model SCN to be less than 10 days, where *τ*_*ent*_ is defined to be the time needed for the cell population to reestablishes 95% of the phase coherence it had before the external forcing was given. We believe *τ*_*ent*_<10 days is a minimal requirement according to many earlier laboratory experiments conducted with rodents [47]. With this additional requirement, our archive reduces further down to 889 networks.

The second additional property that we require is related to the number of phase singularities generated by a finite-amplitude, homogeneous perturbation, and their time evolution. We (BJ and KJL) earlier demonstrated that a global homogeneous perturbation given at an appropriate circadian phase can produce phase singularities about each of which a pinwheel-like phase wave is organized [9]. We have conducted a similar global perturbation experiment on the selected 889 model networks and quantified the properties of phase singularities created (see S3 Fig). Then, we require a successful SCN model network to satisfy: 1) 10 < $N_{\mathrm{ps}}^{\max}$ < 50 and 2) the range of decay time constant for the total number of phase singularities 1.5 < *τ*_*ps*_ < 5.0 days. The requirement has a rather generous range, since there are only a very few experimental measurements on these issues, one of which, yielding $N_{\mathrm{ps}}^{\max}$ = 20 and *τ*_*ps*_ = 3 days, is discussed in S2 Fig. With this additional set of two requirements, our archive of model networks further reduces to 482 cases.

#### Properties of the model SCN networks in the final list

**Fig 7:**
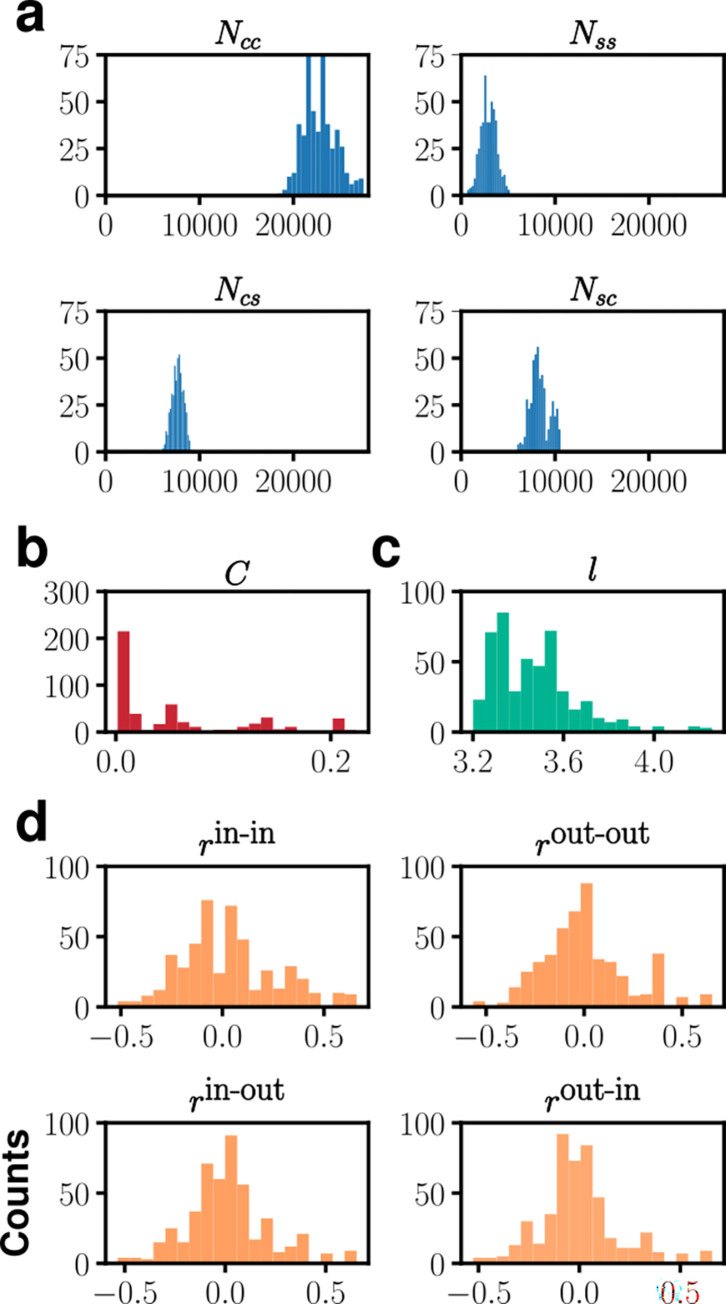
**Histograms of various measures of the selected set of 482 model networks:** (a) number of inter- and intra-sub divisional edges, (b) average clustering coefficient, (c) average shortest path length measured by the minimum number of edges between two nodes, and (d) assortativity coefficients.

Based on the finally selected 482 model SCN networks, we have constructed histograms of various graph-theoretical measures in Fig 7. Most notably, first, the number of intra-connection edges within the core *N*_*cc*_ (mean, 22,500) is, on average, 7.5 times larger than *N*_*ss*_ (mean, 3,000) of the shell (see Fig 7A), while the number of nodes in the shell (2,300) is not too different from that (2,700) in the core. *N*_*ss*_ (mean, 3,000) is even significantly smaller than *N*_*cs*_ (mean, 7,700) and *N*_*sc*_ (mean, 8,400). Nevertheless, *N*_*ss*_ still is an important factor: Most model networks having *N*_*ss*_ < 1,000 do not pass the set of requirements resulting in the final 482 cases. Second, the selected model networks in the final list, in general, do not have a strong tendency to cluster (see Fig 7B). Third, the average shortest path length *l* = 3.47 ± 0.18 (see Fig 7C). Fourth, all four different assortativities (*r*^in-in^, *r*^out-out^, *r*^in-out^, *r*^out-in^) are broadly distributed about 0 (see Fig 7D).

**Fig 8:**
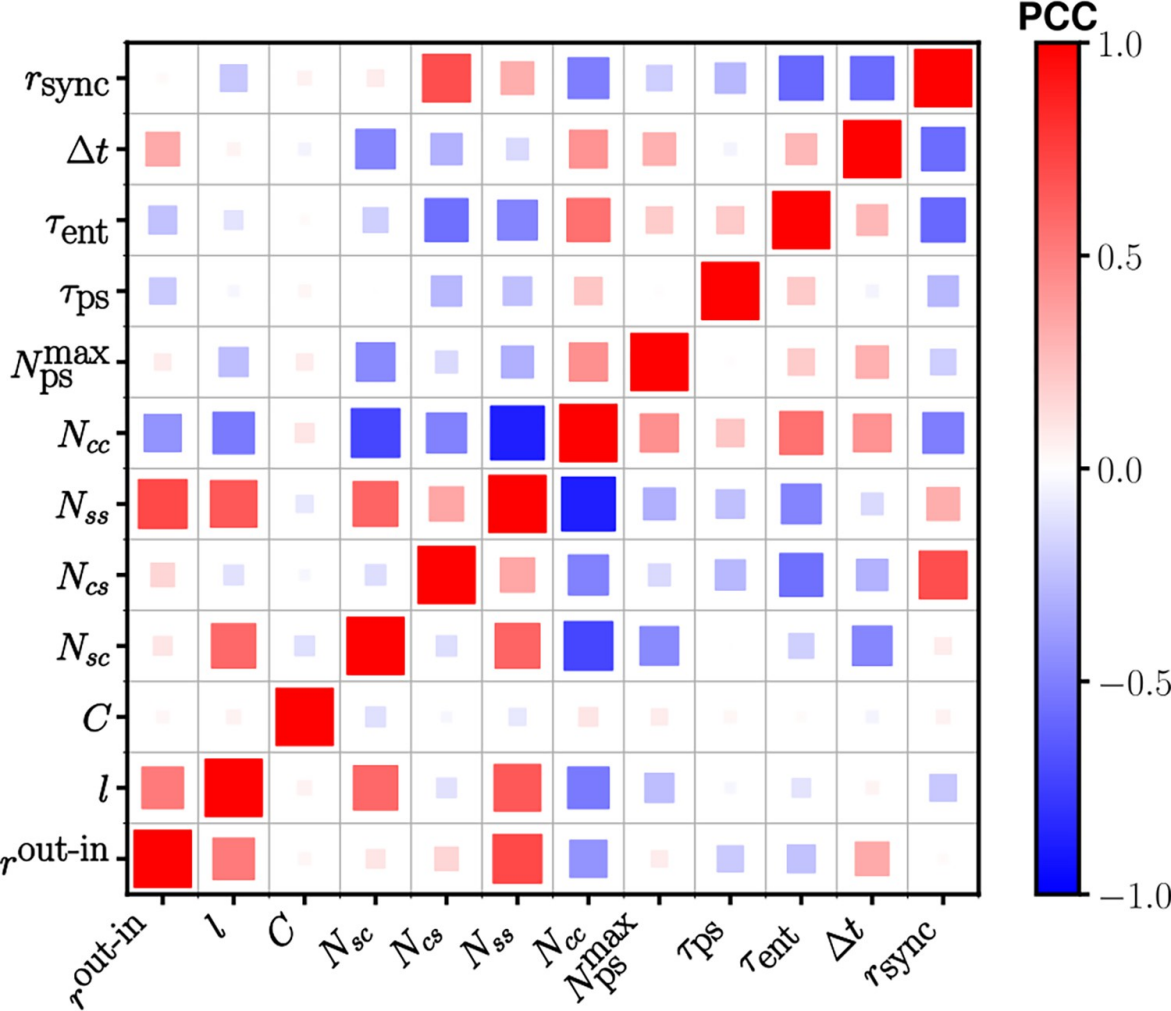
Pairwise Pearson correlation (PPC) map of various network morphological properties and measures characterizing the dynamical aspects of the chosen 482 model SCNs. The number of edges within the core (*N*_*cc*_), within the shell (*N*_*ss*_), from the core to the shell (*N*_*cs*_), and from the shell to the core (*N*_*sc*_) are compared with several different measures related to the circadian phase dynamics within the model SCN.

The basic network properties such as *N*_*cc*_, *N*_*ss*_, *N*_*cs*_, *N*_*sc*_, *l*, all are crucial in determining the properties of SCN circadian waves, and the pairwise correlation map shown in Fig 8 summarizes how they affect the properties related to circadian wave dynamics such as *r*_*sync*_, Δ*t*, *τ*_*ent*_ and $N_{\mathrm{ps}}^{\max}$. First, a positive (negative) correlation between *r*_*sync*_ and *N*_*cs*_ (*N*_*cc*_) exists (see S4A Fig showing how *r*_*sync*_, on average, gradually increases as *N*_*cc*_ decreases for the selected 482 model networks). This is quite natural, since the larger *N*_*cs*_ is the better the core signal gets transmitted to the shell, thereby the more the level of overall phase synchrony is increased. Interestingly, however, the correlation between *N*_*sc*_ and *r*_*sync*_ is very low, even though *N*_*sc*_ ≈ *N*_*cs*_ (see Fig 6B). Second, *τ*_*ent*_ decreases as *N*_*cs*_ increases (or *N*_*cc*_ decreases); again, the larger *N*_*cs*_ confers a more efficient transfer of the phase information from the core to the shell when the external forcing is given only to the core region. Third, increasing either *N*_*cs*_ or *N*_*sc*_ would be decreasing the overall core/shell phase different Δ*t*; interestingly, Δ*t* depends more sensitively on *N*_*sc*_ than *N*_*cs*_. Fourth, although not so significant, networks having a long *l* (over a range of 3 ~ 4 nodes) will give a low *r*_*sync*_ (see Figs 8 and S4A); this is not surprising since an increase in *l* could mean a decrease in the efficiency of connectivity and thus the level of synchrony, and this feature was also pointed out in an earlier study by others employing a network of Kuramoto oscillators [48, 49]. Fig 8 (and S4A) also clearly illustrates the negative Pearson correlation between *N*_*cc*_ and *l*.

Our pairwise correlation analysis suggests that *r*^out-in^, which is found to be widely dispersed over the selected 482 model networks (Fig 7D), seem to have only a minor role; the other three assortativities, *r*^in-in^, *r*^out-out^ and *r*^in-out^, which are on average positively correlated with *r*^out-in^ for the case of positive indegree-outdegree cases (see Sec. 6 in S1 Text), are very much the same as *r*^out-in^ (not shown). One notable feature is that *r*^out-in^ and Δ*t* are positively correlated (see Fig 8). This positive correlation seems consistent with our finding that the larger assortativity is the higher the two subdivisional (core/shell) modularity score *Q*_*cs*_ (see S4B Fig); of course, we would expect a larger Δ*t* for a higher modularity score as it means a weaker inter-subdivisional connection. This positive correlation is also consistent with the finding that the assortativities are positively correlated with *l* (see Fig 8). Except for Δ*t*, the assortativities have a negligible level of correlation with all the other dynamic measures: For example, *r*_*sync*_ has no correlation at all with *r*^out-in^. This is in contrast with general perception that disassortative networks have higher level of phase synchrony than assortative networks [50, 51]. In our cases, increasing *r*^out-in^ means decreasing *N*_*cc*_ and increasing *N*_*ss*_, which would correspond to decreasing (increasing) the level of synchrony in the core (shell), respectively. Then, due to these two opposing effects, a change in *r*^out-in^ (or any other assortativities) would barely change the overall level of phase synchrony for the entire system; in a way, the existence of two different SCN subregions has compromised the potential effect of network assortativities. Another important graph-theoretical measure, clustering coefficient *C*, does not have a meaningful pair-wise correlation with any of all those measures that we have considered for characterizing either the network morphology or the wave dynamics; we believe that this is mainly because the levels of clustering for all selected model networks are rather too small as shown in Fig 7B.

Finally, we comment on the correlations among the measures which characterize the dynamical aspects of the circadian waves. First, for an obvious reason *r*_*sync*_ is negatively correlated with Δ*t*: after all, *r*_*sync*_ measures the level of phase dispersal and Δ*t* is the overall phase difference between the core and the shell. Second, *r*_*sync*_ is also negatively correlated with *τ*_*ent*_. In our model, the external entrainment signal couples directly only with the core region, therefore if the coupling strength between the core and the shell (i.e., *N*_*cs*_) is low (and consequently *r*_*sync*_ is low), the whole system will take longer time (*τ*_*ent*_) to be entrained. Third, *r*_*sync*_ is also negatively correlated with τ_ps_. The origin of this negative correlation is less obvious. The wider phase dispersed a given system (in order words, the larger Δ*t* or the smaller *r*_*sync*_) is, the larger number of phase singularities ($N_{\mathrm{ps}}^{\max}$) being produced (see Fig 8), yet there is not much of correlation between $N_{\mathrm{ps}}^{\max}$ and τ_ps_.

#### One final validation of the selected set of model networks

**Fig 9:**
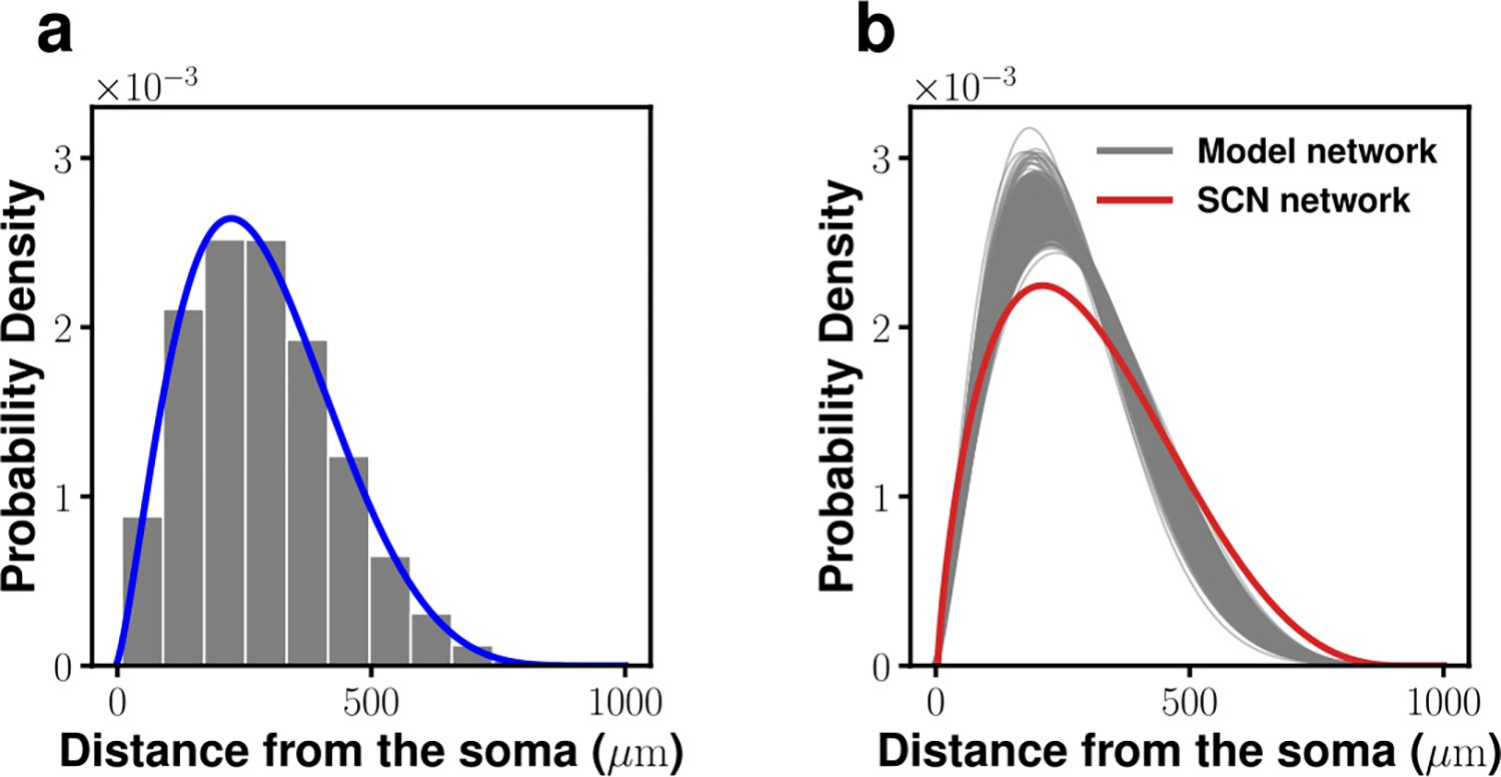
Postsynaptic-to-presynaptic distance *pdf*s. (a) One exemplary case *α* = 0, *β* = 1, *c*_1_ = 0.125, *c*_2_ = 0.5, *c*_3_ = 0.375, *c*_4_ = 0 (blue line: fit to a beta *pdf*). (b) *Pdf*s for the qualified 482 model SCN networks (gray) and an experimentally acquired *pdf* (see Fig 2B).

Finally, Fig 9A plots the probability density functions of the physical distances among all afferent connections for a specific case and Fig 9B show a whole family of them. Very similar to that of experimental measurements (solid red line in Fig 9B), afferent connections from near neighborhood are very rare and the most dominant afferent connections are from the nodes about (average) 209.7 *μm* away. The similarity between the family of curves in Fig 9B and the fitting curve of Fig 2B (or the solid red line in Fig 9B), whose average peak position is 210.1 *μm*, is rather striking; thus, be considered as a good confirmation of our selected network models.

#### Summary and discussion

The journey we have taken so far in this work is about building a (group of) realistic SCN clock cell network(s). It began with very little amount of experimentally acquired information about the real SCN network properties, which, by the way, was obtained through very difficult and time-consuming optogenetic mapping experiments. So, our strategy was, 1) start building a comprehensive archive of dynamic SCN model networks incorporating those measured network properties of (rat and mouse) SCNs, and then 2) utilize experimentally identified circadian phase wave properties as a multi-dimensional gauge of the model networks’ fitness. Concerning the first part of our strategy, so far, very few existing mathematical model SCNs had incorporated an actual experimental data related to any SCN networks. Perhaps more important is second part: using the features of collective dynamics (i.e., circadian phase waves) as a guide for deciphering the SCN clock cell network properties. This is not a completely new idea (see reference [16]) but has not been explored much. After all, a big part of neuroscience in general is to correlate neuronal population dynamics with the structure of its underlying neural network.

There are three important parameters in our model SCN: *D*, *ω*, and *K*. The parameter *D* sets the strength of diffusive coupling, and in this study, we could make a very good estimate of it based on the coherence length of the circadian phase waves of TTX-treated SCN samples. The parameter *ω* controls the circadian period of the model oscillator, and the range of *ω* was reasonably set according to the earlier reports on the inhomogeneous oscillatory nature of the SCN clock cells [5]. We have used a particular value of *K* = 1.5×10^−2^ which would provide the maximum number of potential model networks satisfying the three mandatory conditions we imposed. Perhaps, one important drawback of our current network model(s) is that we have used a grand unified coupling strength *K* for all AP-mediated functional cell-to-cell couplings. Obviously, this assumption is not realistic; and in the future we are to explore some exemplary cases in which a range of dispersed *K*s is allowed and *K*s are distributed (in many different ways) over all the edges and see how that would change the conclusion of current paper.

As a validation for a successful SCN model network, we have required the following properties: 1) the level of overall phase synchrony of *r*_*sync*_>0.9; 2) the direction of phase wave propagation is from the shell to core and the phase dispersal Δ*t*>1 hr; 3) the resynchronization time after a global perturbation is shorter for the core than the shell (Δ*τ*_*rec*_>0); 4) phase-resetting in response to a sudden 6-hr phase-shifting external forcing completes within 10 days; and lastly 5) the number of phase-singularities induced by a global perturbation and the decay time constant of them satisfy (10 < $N_{\mathrm{ps}}^{\max}$ < 50, 1.5<*τ*_*ps*_<5.0 days).

The followings are some key properties of the qualified SCN model networks:

1. Indegrees must be positively correlated with outdegrees.
2. The number of intra-core edges (*N*_*cc*_) is about 7.5 times that of intra-shell edges (*N*_*ss*_).
3. *N*_*cs*_ is positively (negatively) correlated with *r*_*sync*_, τ_ent_, and $N_{\mathrm{ps}}^{\max}$.
4. Naturally, as *l* increases *r*_*sync*_ drops.
5. As the degree assortativities increase, the core/shell subdivisional modularity score increases and so does Δ*t*.
6. The clustering coefficient *C* seems not important for any dynamic feature of the SCN circadian waves.
7. The distance *pdf* for the afferent connections fits well to a beta distribution function and is very consistent with that of experimentally measured one.

The in-out degree relation greatly affects the dynamics as well as the structure of the given network. In our setting of the model SCNs, if the indegree and outdegree are negatively correlated to one another, the inter-subdivisional (i.e., core-to-shell and shell-to-core) edges will be more dominant over the intra-subdivisional (core-to-core and shell-to-shell) edges. Moreover, the shortest path lengths (*l*s) are generally much smaller for the negatively correlated cases as compared to those of the positively correlated cases (see S5 Fig); accordingly, Δ*t*s are smaller in these cases with a better system-wise synchronization. In other words, the negatively correlated systems favor a globally synchronized oscillation over a propagating circadian phase wave. So, the cases of negative indegree-outdegree correlation are considered unrealistic, thus, excluded.

Alongside the fact that the in-out degree relation should be positive, the edge distribution is to be rather heterogeneous: The intra-core edge connections *N*_*cc*_ is way higher than *N*_*ss*_, *N*_*cs*_, and *N*_*sc*_. And this property not only makes shell-to-core propagating circadian waves possible, but also confers a faster recovery for the core than the shell when the system was disrupted by a global perturbation. Also, we have confirmed that the opposite cases (i.e., *N*_*ss*_ > *N*_*cc*_) cannot meet the two requirements. This finding is consistent with the result of Abel *et al*., who analyzed SCN network morphology based on the sequences of experimentally obtained PER2::LUC images [15], and that of Taylor *et al*. [36], who took a similar approach of testing multiple network configurations and selecting models that reproduced experimental phase dynamics. Of course, an excessively high *N*_*cc*_ would mean too small number of inter-subdivisional edges (*N*_*cs*_ and *N*_*sc*_), which will not only hamper the system-wise phase coherence but also prevent a fast adaptation to external stimuli. Consequently, an ideal ratio of *N*_*cc*_/*N*_*ss*_ was found to be, on average, 7.5.

It is known from many earlier studies that “small-world networks,” which have a relatively high *C* and a low *l* compared to the random network, generally show a high level of phase synchrony [52, 53]. And several previous modelling studies [25, 54, 55] have considered small-world networks as a basis for modeling SCN circadian dynamics. On the other hand, the validated SCN model networks of our final list are not a small-world network, as their *C*s are rather very small (< 0.1); that is, *C* does not play a significant role in the SCN model networks. The shortest path length *l*, however, has a noticeable effect on the properties of circadian waves supported by our validated models. As for the level of synchronization in a “scale-free network,” the role of *l* (*C*) is more significant for high (low) coupling strength [49]. In this regard (although our model networks are not a scale-free network), we may view the chosen coupling strength *K* (= 1.5×10^−2^) is high enough that a good level of synchronization (*r*_sync_ > 0.9) is achieved. Again, all the validated SCN model networks of ours support an exponential distribution not only for the indegree but also for outdegree and total-degree, as 1) we have assumed an exponential indegree distribution from the beginning (based on our earlier experimental measurements) and 2) indegrees and outdegree must be positively correlated.

The network assortativity (or disassortativity) is known as an important factor affecting the level of synchrony and in general disassortative networks show a higher level of synchrony over assortative networks [50, 51]. For the validated SCN model networks, however, all four assortativity coefficients do not affect the level of synchrony *r*_*sync*_ in any significant way. That is because assortativitity coefficients are negatively (positively) correlated with *N*_*cc*_ (*N*_*ss*_) as shown in Fig 8; that is, increasing assortativity decreases (increases) the level of synchrony in the core (shell). As for the whole SCN, these two opposing effects cancel one another, thus there will be not much of change in *r*_*sync*_. In the meanwhile, an increase of assortativity makes the network more modularized into the core and shell more clearly (see S4B Fig); and it seems to be the main reason why assortativity is positively correlated with the average phase difference Δ*t* between the core and shell (see Fig 8). After all, the existence of two subregions (core and shell) within the SCN, which was often considered a priori in previous SCN modelling studies [16, 25, 56], turns out to be a good assumption. In addition, an increase of assortativity reduces the difference between *N*_*cc*_ and *N*_*ss*_, thus, makes the system more quickly adapt to an external forcing (i.e., *τ*_*ent*_ decreases) or recover faster from a (phase singularity generating) global perturbation (i.e., *τ*_*ps*_ also decreases).

In a recent paper [57] Wang *et al*. has proposed a challenging idea with which one can solve some difficult “inverse problems,” for example, estimating all different coupling functions mediating all node-nodes as well as those for the local dynamic elements, entirely based on experimental image time series. As a proof of concept, the authors applied their method to an experimental data acquired by Abel *et al*. [15], which is bioluminescence PER2 expression time series of ~ 500 cells within a pair of nuclei. But, to make their test simpler, the authors had presumed a weakly phase-coupled linear oscillators as a model “framework”: In other words, the local kinetic functions were assumed to be constants (angular frequencies) and the coupling functions were a “truncated Fourier harmonic series” of phase differences *ϕ*_*i*_−*ϕ*_*j*_, where *ϕ*_*i*_ and *ϕ*_*j*_ are the phases of the *i*th and *j*th oscillator, respectively. A “coupling strength” *α*_*ij*_ between two cells (labelled by *i* and *j*) was defined with the relevant Fourier coefficients and a measure called “total outgoing strength (∑_*j*_*α*_*ij*_)” was evaluated for each cell. They claimed that 1) 5% of all (~ 500) recorded cells could be considered as hubs, and they were dispersed randomly and equally to both nuclei forming a pair; 2) a wide (~10 fold) scatter of total outgoing strength for the SCN oscillators; 3) widely varying (complex harmonic) coupling functions. Although we cannot comment on the result 3), their results 1) and 2) seem to be consistent with one of our claims that both outdegree and indegree would exhibit an exponential distribution. However, Wang’s paper has no detailed analysis concerning graph-theoretical measures of the SCN clock cell network morphology.

Finally, we should comment on the significant and excellent modeling efforts by others in the past for various aspects of the mammalian SCNs; they have formed the foundation of the current work. Schmal *et al*. [27] used Poincaré oscillators as for the individual oscillators (as in our model) but employed a global mean-field cell-cell coupling to discuss the application of TTX imparting a reduction in the strength of global coupling constant. In the meanwhile, Unosson *et al*. [58] took a rather different approach, assuming a population of cells that has no explicit intercellular communication but effectively coupled by “multiplicative random effects with a first order Markov random field prior distribution,” which is conceptually akin to the diffusion-mediated coupling in our model. Based on the inferred dynamic properties of the cells, they could distinguish two different subpopulations, the central core in which the rhythm is resilient and the outer shell which is more easily affected by external stimuli. Spencer *et al*. [59] brought up with an interesting idea that the SCN network topology could have evolved evolutionally for the cost effectiveness. They found a sparser SCN network topology is less costly for maintaining the synchrony of the clock cell population. Their study was based on a weekly coupled phase model of Kuramoto. Very recently, Zhou *et al*. [60] explored the effects of dynamic (or plastic) network connectivity on the SCN circadian rhythm assuming three (random, scale-free, and small-word) different types of linear couplings. Plastic coupling strength is an element that is not incorporated into but can be added on top of our current model. It would be interesting to see how the plasticity impact on the various measures of SCN phase waves. Myung and Pauls [61] studied a hybrid SCN network model in which cell-cell coupling have two opposing types, phase synchronizing (“attractive”) and phase desynchronizing (“repulsive”), which render the oscillators to organize into two different groups. It was then claimed that seasonal photo-period variation can be reflected into the phase relation of the two subpopulations. Years earlier, Bodenstein *et al*. [62] hypothesized a similar idea that the change not in the individual oscillators but the structure of the SCN network is responsible for the seasonal SCN circadian dynamic changes. Unlike the model of Myung and Pauls, Bodenstein *et al*. assumed a linear coupling as in our Eq 2 but modulated the percentage of long-range cell-cell connections between the core and the shell to create experimentally observed seasonal circadian dynamic changes. In the same light, it will be interesting to see how varying the photo-period in the external forcing term of our model alone (without altering the network structure) would change the dynamic properties of the SCN phase waves.

## Conclusion

The SCN clock cells employ at least two different cell-to-cell coupling mechanisms: One is local diffusive coupling among adjacent cells and the other is potentially, far-reaching, extensive, AP-mediated coupling. In this study, the strength *D* of the diffusive coupling could be estimated based on careful analysis on the properties of turbulent phase wave activities of TTX-treated SCNs in experiments, in which no APs were allowed. However, there was no direct way to determine the strength *K* of the AP-mediated cell-to-cell coupling. In fact, the challenge was to estimate the strength *K* as well as the morphology of the AP-mediated SCN clock cell connections all together. So, we set out an exhaustive scan of *K* values and various network morphologies; we have found a suitable value of *K* and network morphologies that render the mathematical model system to support circadian phase waves whose properties closely match those of real SCN phase waves observed in experiments. The most prominent properties of the group of validated networks are that 1) indegree and outdegree correlate positively; 2) the number of intra cell-cell connections inside the core is several times higher than that inside the shell; 3) the selected SCN model networks are not a small-world network in the sense that their clustering coefficients *C*s are very small and there are no “hubs” (i.e., the degree distribution has no fat tail); and 4) all dynamic properties of (perturbed as well as autonomous) SCN circadian waves are consistent with the picture of the SCN having two well modularized subregions (core and shell). We believe that our current findings would shed new light and give a guide for future experimental explorations on the very much unexplored AP-mediated clock cell network morphology within the SCN.

## Methods

### Ethics statement

The animal experiments discussed in Figs 1 and 2 in this study were approved by our institutional review board for animal research, the Korea University Institutional Animal Care and Use Committee (KUIACUC-20170405-2).

Below, we briefly describe several graph-theoretical measures that are used in this paper.

### Clustering coefficient

The propensity of a network to form tightly connected neighborhoods, compared to the random uncorrelated case, is measured by the clustering coefficient *C* [63]. For a directed network, *C* is defined as:

$$C=\frac{1}{N}\sum_{i=1}^{N}\frac{2T_{i}}{k_{i}^{\mathrm{tot}}(k_{i}^{\mathrm{tot}}-1)},$$ (3)

where *N* is number of nodes, *T*_*i*_ is the number of triangles through node *i* and $k_{i}^{\mathrm{tot}}$ is the total degree of node *i*. There are no reciprocal connections since we excluded bidirectional edges. To calculate *C*, we used a Python package, NETWORKX.

#### Assortativity coefficients

Assortativity coefficient r measures the preference of nodes to make a partner from the same group as themselves, and it is defined as:

$$r=\frac{\sum jk(e_{jk}-q_{j}^{s}q_{k}^{t})}{\sigma_{s}\sigma_{t}},$$ (4)

where *e*_*jk*_ is the joint probability matrix representing the probability of selecting an edge with *j* degree at the source node (*s*) and *k* degree at the target node (*t*) when randomly picking an edge. When *p*_*k*_ is the probability that a node has *k* degree, *q*_*k*_ is defined as:

$$q_{k}=\frac{(k+1)p_{k+1}}{\sum kp_{k}}.$$ (5)

If $q_{j}^{s}$ and $q_{k}^{t}$ are independent, then $e_{jk}=q_{j}^{s}q_{k}^{t}$, therefore *r* = 0. *σ*_*s*_ and *σ*_*t*_ are the standard deviations of $q_{j}^{s}$ and $q_{k}^{t}$, respectively. [64] To calculate the degree assortativity coefficients, we used a Python package, NETWORKX.

### Average shortest path length

The shortest path length is the minimum number of edges between nodes. The average shortest path length is defined as:

$$l=\sum_{s,t\in V}\frac{d(s,t)}{N(N-1)},$$ (6)

where *N* is number of nodes, *V* is the set of nodes, and *d*(*s*, *t*) is the shortest path from *s* to *t*.

To calculate the degree assortativity coefficients, we used a Python package, NETWORKX.

### Modularity and community detections

Modularity is an index indicating the degree of structural division of sub-networks within the network and is defined as follows.

$$Q_{cs}=\frac{1}{2m}\sum_{ij}\left(A_{ij}-\gamma \frac{k_{i}k_{j}}{2m}\right)\delta (c_{i},c_{j}),$$ (7)

where *A*_*ij*_ is the adjacency matrix of the network, m is the number of edges, *k*_*i*_ is the degree of *i*th node, and *γ* is the ‘resolution parameter.’ *δ*(*c*_*i*_, *c*_*j*_) is 1 if i and j are both in the core (or the shell), otherwise 0. To automatically find sub-networks (or communities), we performed community detection using the Leiden algorithm [65]. In this case, we set the resolution parameter *γ* = 1.0, which shows consistency in detection [66]. To calculate modularity and detect modularized communities, we used a Python package, IGRAPH.

## Supporting information

S1 Text Building an extensive archive of SCN network models and additional reference information. (DOCX)

S1 Fig Circadian phase waves supported by mouse SCNs. 2D snapshots of circadian phase maps (all images are reoriented such that the midline of the third ventricle (3V) aligns along the y-axis). The direction of each white arrowhead depicts the average instantaneous direction of phase wave propagation. Different phase maps correspond to a different organotypic *ex vivo* mouse SCN (coronal section) culture, and they are taken at different days (and hours)-in-vitro (DIV). The exact geophysical times when the phase maps were acquired are unknown. The SCN slice cultures are quasi-3D (with a typical thickness of 100 ~ 150 *μm*); therefore, shown are 2D projection images of 3D samples. The frame #1 corresponds to Fig 1A. (The original PER2::LUC signal data were shared at https://github.com/johnabel/scn-resynchronization-data-2016).
(TIFF)

S2 Fig Creation of phase singularities and their spatiotemporal evolution. (a) Snapshot circadian PER2::LUC phase maps, just before (left) and 4 hr after (right) a temperature shock (TS) was delivered. The colored dots in (a) mark the positions of all phase singularities at the star-marked time point in (c). Phase singularities are identified by employing an Iyer-Gray method [67]. (b) Temporal traces of all phase singularities, from their births to deaths. A Crocker-Grier linking algorithm [68] is used for linking a set of different singularities identified at different time points. (c) Temporal evolution of the total number of phase singularities. The lifetime of the perturbation-induced phase singularities is about 3 days. The first (second) temperature shock pulse is given at t = 3.3 (9.6) days; the time points of the two heat shocks are marked by two red vertical lines. We point out that a timely perturbation is very critical for the creation of phase singularities: For instance, the second perturbation given approximately at day 9.6 has failed to produce any phase singularities. [Our (BJ and KJL) earlier experimental data published in Ref. [9] is used for this new analysis.]
(TIFF)

S3 FigMaximum number of phase singularities ($N_{\mathrm{ps}}^{\max}$) generated by a homogeneous perturbation given at various circadian phases ϕ (top) and population mean PRC (bottom).As for the perturbation, 0.7×*r*_*i*_ where $r_{i}=\sqrt{x_{i}^{2}+y_{i}^{2}}$, is subtracted from every node *I* at a single circadian instance in order to mimic the decrease in the level of PER2::LUC subject to a temperature pulse shock [9]. The top frame includes 3,500 lines, each of which corresponds to a randomly selected network (having r_sync_>0.9) of our SCN model archive. Note that for all cases $N_{\mathrm{ps}}^{\max}$ peaks around the same unstable fixed point (marked by a red dot) of the PRC. Δ*ϕ* is the amount of mean (over 3,500 cases) phase shift due to the homogeneous perturbation and the error bars represent the standard deviation of Δ*ϕ*.(TIFF)

S4 Fig **The level of phase synchrony in (a) and average phase difference between the core and shell (b) as a function of various network properties.** *Q*_*cs*_ is the modularity score based on two modules (core and shell).
(TIFF)

S5 FigThe average shortest path lengths for different indegree-outdegree relations for all the SCN model networks considered in Table 1.Note that $\bar{l}$ does not change much for networks having a positive indegree-outdegree relation.(TIFF)

S1 Movie Three exemplary circadian waves supported by mouse SCNs (EXPERIMENT). See Fig 1 (and S1 Fig) for details.
(MOV)

S2 Movie Phase waves in a steady state created by a validated model SCN (SIMULATION). For this particular case *r*_*sync*_ = 0.95 and Δ*t* = 1.7 hr.
(MOV)

S3 Movie Evolution of phase singularities created by a perturbation (EXPERIMENT). See Fig 3 for details. Time (text) colors are, BLACK (before any perturbations), YELLOW (after first perturbation), and RED (after second perturbation).
(MOV)

S4 MovieEvolution of phase singularities created by a perturbation (SIMULATION).The perturbation was delivered when the phase distribution was passing through the unstable fixed point of PRC. Right after the perturbation, *N*_*ps*_ initially increases reaching $N_{ps}^{max}$ = 40 but soon decays with *τ*_*ps*_ = 3.5 days. The time index changes its color from black to red after the perturbation is given.(MOV)

S5 Movie SCN phase waves subject to a ‘light-dark’ modulation (SIMULATION). For this particular example we measure *τ*_*ent*_ = 4.5 days.
(MOV)
