# Supplementary material for: Deciphering clock cell network morphology within the biological master clock, suprachiasmatic nucleus: From the perspective of circadian wave dynamics
Source: PLoS Comput Biol. 2022 Jun 6;18(6):e1010213. doi: 10.1371/journal.pcbi.1010213 (PMC9203024; doi:10.1371/journal.pcbi.1010213)
Supplement: S1 Text — (DOCX) [file pcbi.1010213.s001.docx]

**S1 Text**

for

Deciphering clock cell network morphology within the biological master clock, suprachiasmatic nucleus: From the perspective of circadian wave dynamics

Hyun Kim^1^, Cheolhong Min^1^, Byeongha Jeong^2^, and Kyoung J. Lee^1^*

**1. Generating 3 major categories of SCN model networks based on** $\boldsymbol{k}^{\boldsymbol{in}}\boldsymbol{-}\boldsymbol{k}^{\boldsymbol{out}}$ **correlation**

For generating our archive of model SCN networks, we have considered 5 (3) different positive (negative) correlation levels and 1 randomly correlated case as schematically illustrated in Fig Aa. Here, the different level of correlation, more or less, refers to a different overall ratio between the outdegree and indegree of a node up to a certain value of indegree: For example, for the case of pos(50) the ratio is approximately 1 for the entire range of indegree k^in^; but for the case of pos(10) it is about 1.4 only up to k^in^ ~ 10, beyond which k^out^ scatters randomly around 14.5. The existence of this critical k^in^ ~ 10 is unavoidable since the ratio of 1.4 cannot be supported for the entire range of k^in^ as the total sum of k^out^, which is the same as the total sum of k^in^, is a fixed number. For the three cases having a negative correlation, the slopes are chosen to be stiffer to avoid fragmented network; consequently, many nodes have very small value (~ 0) of k^out^ for the inversely correlated cases. We have used a beta cumulative distribution function $B\left( 1,1 \right)$ for the positive cases (see Fig Ab) and $1-B\left( 5,20 \right)$ for the negative cases (see Fig Ac).

**
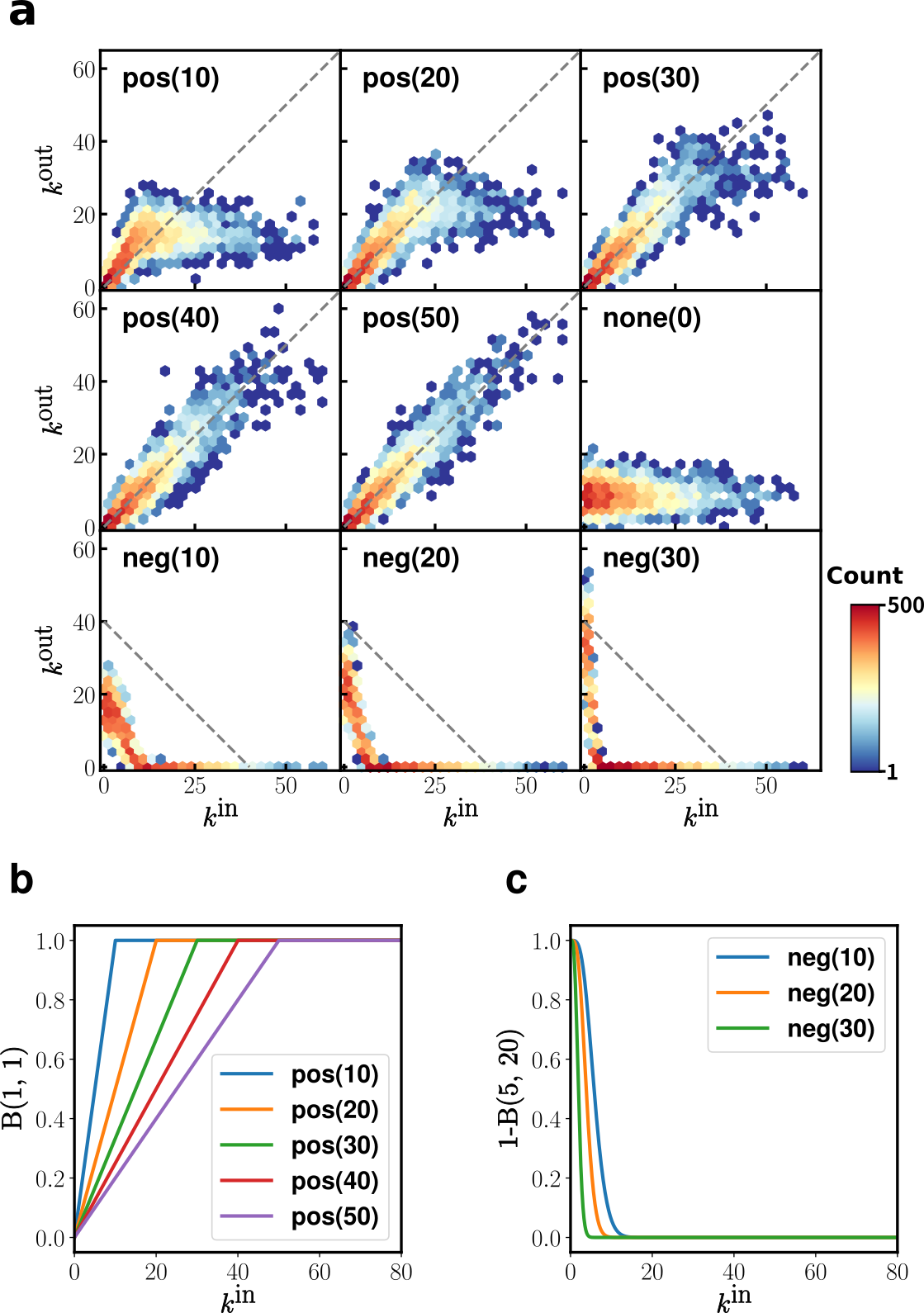
**

**Fig A. Nine different types of outdegree vs. indegree correlations.** (a) positive (prop10~50), none, and negative (inv10~30) cases. (b) Beta cumulative distribution functions B(α=1, β=1) of with k^in, max^ = 10 (blue), 20 (orange), 30 (green), 40 (red), and 50 (violet), used for the five positive correlations in (a). (c) 1–B(α=5, β=20) of , where k^in, max^ = 10 (blue), 20 (orange), and 30 (green), used for the three negative correlations in (a).

**2. Generating a network maximizing its clustering coefficient for a given populations of nodes**

As a simple way to build a network to have a large clustering coefficient, on average, we first arrange all the (indexed) nodes along a circle on a 2D space in a random order as illustrated in Fig B (left); second, make edge connections of a given node, one by one, preferentially to its (physically) near neighbors and do the same for every node along the circle. To implement this proximity-based edge connection scheme systematically, we compute a distance matrix *D^d^*, whose element *d_ij_* is the physical distance between *i*th node and *j*th node on the *x-y* plane. Then, set the edge connection (between *i*th node and *j*th node) probability to be the value of the “horizontally flipped hyperbolic tangent function” of *d_ij_* (Fig B, right). We name the matrix of connection probability to be *P*(*D^d^*): One example is shown in Fig 3b (first thumbnail frame) of the main text. If we connect the nodes according to the probabilities of *P*(*D^d^*) alone, we effectively create a network with a maximum level of clustering coefficient on top of the prescribed indegree-outdegree correlations.


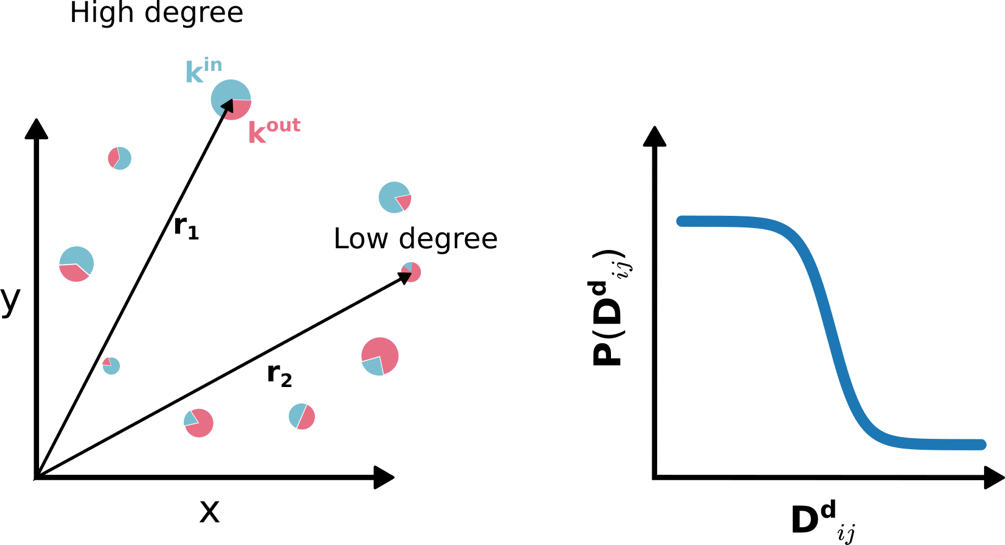


**Fig B. A method for maximizing network clustering coefficient for a given population of nodes.** Left: Nodes are placed randomly along a circle on a 2D space with an equal distance apart from immediate neighbors. Right: Connection probability matrix as a function of physical distance between two nodes lying on the circle.

**3. Generating a network maximizing its assortativity**

In order to achieve a network having the largest (e.g., k^in^ – k^in^) assortativity, we can repeat the process we have gone through for obtaining $D_{ij}^{d}$ and $p_{ij}^{c}$ but this time for D^in-in^ and $p_{ij}^{\text{in-in}}$, assuming the distance matrix element for $D_{ij}^{\text{in-in}}$ is $\Delta k_{ij}^{\text{in-in}}=\left| k_{j}^{\text{in}}-k_{i}^{\text{in}} \right|$, where $k_{j}^{\text{in}}$ and $k_{i}^{\text{in}}$ represent the indegree of jth and that of ith node, respectively. So, the smaller the value of $\Delta k_{ij}^{\text{in-in}}$ is the more likely ith node connects to jth node, thus, increasing the level of indegree/indegree assortativity. We define the corresponding matrix of edge connection probabilities as P(D^in-in^), maximizing indegree/indegree assortativity (second thumbnail in Fig 3b in the main text). Likewise, we can obtain the other edge connection probability matrices, P(D^out-out^), P(D^in-out^), and P(D^out-in^), which would maximize the outdegree/outdegree, indegree/outdegree, and outdegree/indegree assortativity, respectively. The total connection probability matrix $P^{total}$ (see Fig 3b in the main text) is a linear sum of weighted matrices of P(D^d^), P(D^out-out^), P(D^in-out^), and P(D^out-in^), and P(R). P(R) represents a random connection matrix.

**4. Placing nodes within a 2D model nucleus according to their degree values**

We have a problem of mapping the node index to a grid point (p, q), where p and q are the position indices along x- and y-axis in a 2d space, respectively. We assign a single index k to each one of all (p, q)s such that 1) the central position (p^c^, q^c^) of the SCN (marked by a star symbol in Fig 4a) has the maximum k and 2) all other grid points (p, q)s map to k as a decreasing function of their distances to the center (p^c^, q^c^). Likewise, we reorder the node index i in descending order of its corresponding total degree k^tot^ (alternatively, we have also used k^in^ or k^out^), and match the reordered ith node to the kth grid position. Consequently, the larger k^tot^ a node has, the closer the corresponding node to (p^c^, q^c^) becomes (see Fig 4a, left). Alternatively, we can also reorder the node index in ascending order of the total degree k^tot^ and obtain the case having degree low for the core (see Fig 4a, right). We have also considered 5 different levels of randomness in the index k assignments. All together, we have considered 30 (= 2 × 3 × 5) different ways of assigning nodes to the grid space of the model SCN.

**5. Downsizing the SCN model archive uniformly**

After computing all 5 graph-theoretical measures (C, r^in-in^, r^out-out^, r^in-out^, r^out-in^) for all candidate networks, we uniformly under-sample the candidate networks in the 5D metric space of the measures (i.e., one sample per one 5D grid mesh). Fig C shows the reduced set of 5D points projected onto the 2D space of (C, r^in-in^). As the figure well conveys, the case for no (‘none’) or small positive (e.g., ‘pos10’) k^in^ - k^out^ correlation covers a much broader space in the 5D metric space and consequently has a larger number of network candidates than the cases having a larger positive correlation (e.g., ‘pos50’). The number of cases for different types of k^in^ - k^out^ correlation is listed in Table 1 in the main text.

This above downsizing process assumes that two different networks that are very close in the 5-dim space of (C, r^in-in^, r^out-out^, r^in-out^, r^out-in^) would produce a similar spatiotemporal circadian phase dynamics. Closely related to this under-sampling process is the question of reproducibility of the network structure created based on the connection probability matrix $P^{total}$ (see Fig 3b in the main text). Since the network morphology is built probabilistically, even for a fixed set of parameter values of α, β, c_1_, c_2_, c_3_, and c_4_, one would expect a similar but different morphology [i.e., a different 5D point of (C, r^in-in^, r^out-out^, r^in-out^, r^out-in^)] for different trials; yet we have confirmed that the change is small (see Fig Da). Moreover, we also find that two different networks that are close in the 5-dim space of (C, r^in-in^, r^out-out^, r^in-out^, r^out-in^) would produce a similar spatiotemporal circadian phase dynamics (see Fig Db). So, the downsizing process alongside the reproducibility is justified.


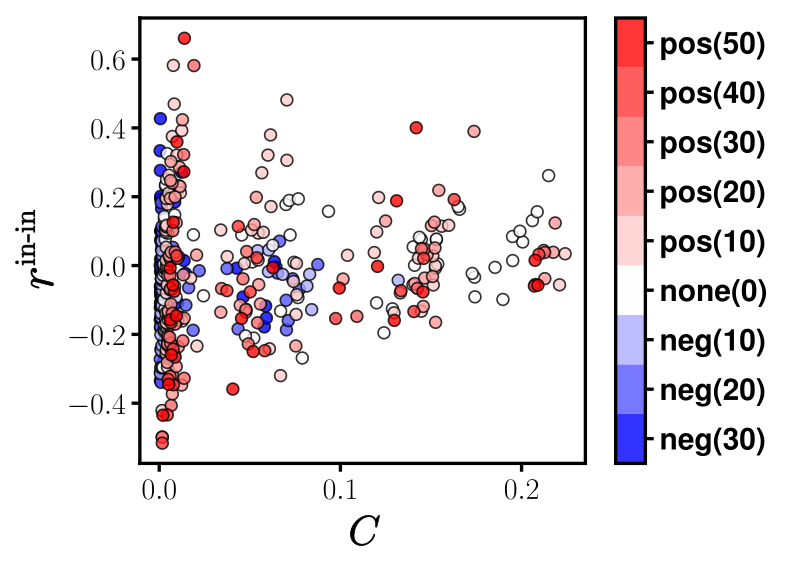


**Fig C.** C **and** r^in-in^ **for the under-sampled pool of candidate networks.** Different colors represent different types and levels of indegree-outdegree correlation (see the color bar). This is a projection of (C, r^in-in^, r^out-out^, r^in-out^, r^out-in^) points on to 2D space of C and r^in-in^ for the under-sampled network archive. (total number of points = 838)

**
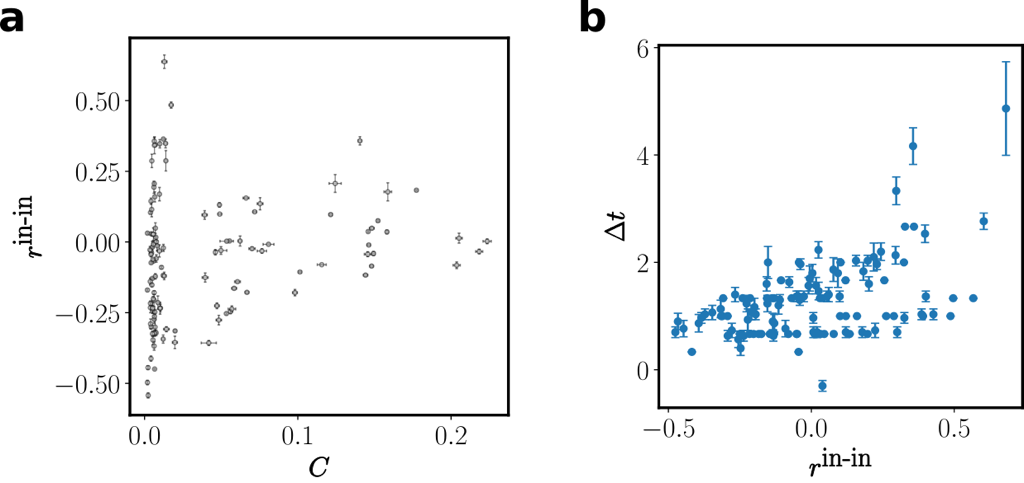
**

**Fig D. Level of variations in different trials.** Shown in (a) are the average values (points) and the associated standard deviations (error bars) of $C$ and $r^{in-in}$ for 10 different trials for each of 120 different connection probability matrices $P^{total}$s (see Fig 3b and its related description in the main text). A different initial condition is used for each different trial. The 120 points are sampled from Fig C, especially, from the cases of positive $k^{in}-k^{out}$ correlation. Since the network morphology is built probabilistically, even for a fixed set of parameter values of α, β, c_1_, c_2_, c_3_, and c_4_, one would expect a different morphology [i.e., (C, r^in-in^, r^out-out^, r^in-out^, r^out-in^)] for different trials; yet we have confirmed that the change is small as shown in (a). Moreover, we also find that two different networks that are close in the 5-dim space of (C, r^in-in^, r^out-out^, r^in-out^, r^out-in^) would produce a similar spatiotemporal circadian phase dynamics: (b) characterizes the average values (points) and their standard deviations (error bars) of the core-shell phase difference $\Delta t$ and $r^{in-in}$.

**6. Different assortativity relationships**

Note that for the cases of a strong positive k^in^ - k^out^ correlation, r^in-in^, r^out-out^, r^in-out^, and r^out-in^ all are, on average, positively correlated each other, since the larger k^in^ a node has the larger k^out^ it will have, and assortativity measures the preference of nodes to make a partner from the same group of nodes having a similar in(out)degree as themselves (see the 1^st^ row of Fig E). For a negatively correlated case, the larger k^in^ a node has the smaller k^out^ it will have (or vice versus); therefore, networks that have a larger r^in-in^ would have a smaller r^in-out^ (r^out-in^) and a larger r^out-out^ (see the 3^rd^ row of Fig 9). Finally, for the weakly (or not) correlated random cases, there is no clear functional relationship among r^in-in^, r^out-out^, r^in-out^, and r^out-in^ (see the 2^nd^ row of Fig E).

**
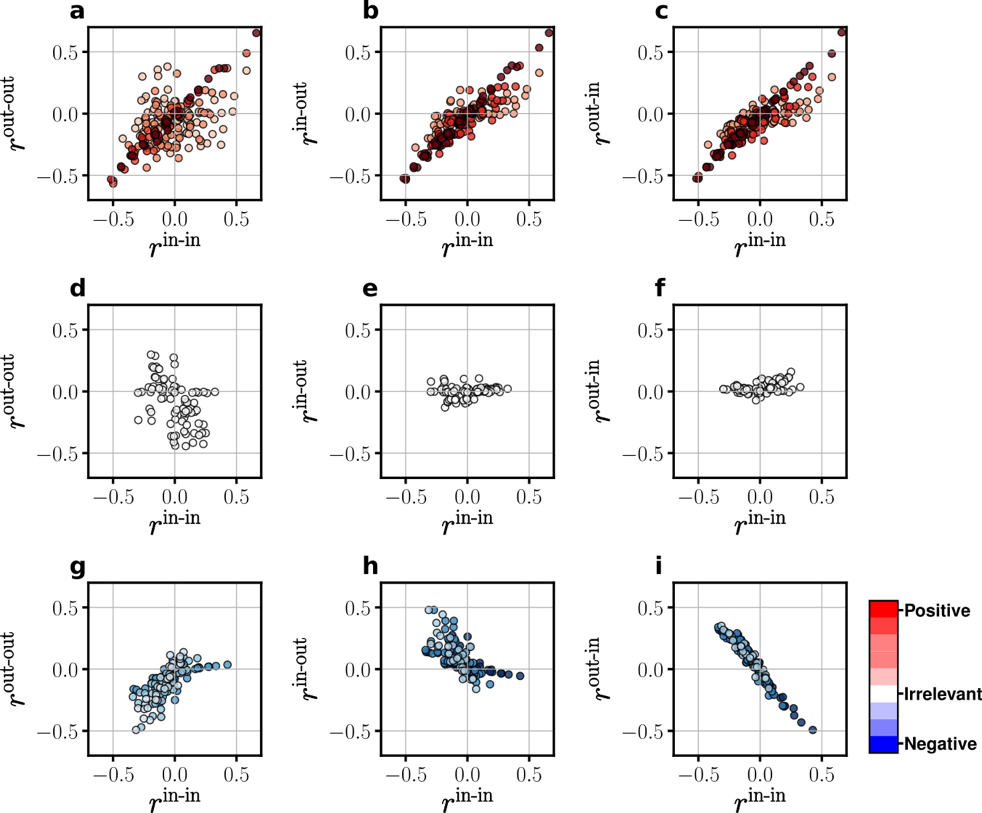
**

**Fig E. Different assortativity relationships for different types of model networks categorized by different** k^in^ - k^out^ **relationships.** k^in^ - k^out^ correlation is positive (colored red) for a, b, c; negative (blue) for g, h, i; and random (white) for d, e, f. Note that the stronger the k^in^ - k^out^ correlation gets, the more evident the assortativity correlation becomes.

**7. Pearson correlations between the number of inter- and intra-subdivisional edges and different assortativity coefficients**

Fig F shows 12 colormaps of Pearson correlation coefficient between the overall network assortativity and the number of (intra- and inter-subdivisional) edges, based on the pool of 25,140 model networks (see Table 1). The goal of this analysis is to set up a reference so that we can compare information about the entire set of networks to the realistic "selected" networks. We first focus on the 6 cases of Fig Ea. The case of k^in^ referenced, ‘Dense Core’ of positively correlated k^in^ and k^out^ (i.e., the map on the 1^st^ column and the 1^st^ row of Fig Fa) is easy to comprehend: Since k^in^ is large for the core, k^out^ is also large for the core; then, both k^in^ and k^out^ will be small for the shell; so, N_cc_ will be large. Subsequently, the larger r^in-in^ gets, the nodes within each subdivision will make more intra-edge and less inter-edge connections; so, r^in-in^ will be positively correlated with N_cc_ (and N_ss_) and be negatively correlated with N_cs_ (and N_sc_). Here we should point out again that for the core-dense case N_cc_ is much larger than N_ss_ (see Fig 4b), therefore in the above heuristic explanation, the role of N_cc_ is more pronounced over that of N_ss_. The similar series of arguments can be used to explain the colormap of the 1^st^ row - 2^nd^ column, ‘Dense Shell’ of Fig Fa. Moreover, since k^in^ and k^out^ are positively correlated for the case of Fig Fa, all 4 assortativities (r^in-in^, r^out-out^, r^in-out^, r^out-in^), which are positively correlated one another (see Fig F), will have a similar correlation with the number of (intra- and inter-subdivision) edges.

The maps shown in Fig Fb correspond to the nodes which have a large (small) k^in^ (k^out^); they are a bit more complex to explain. So, for now we focus on the colormap of the 1^st^ row – 1^st^ column (k^in^ referenced, ‘Dense Core’ case) of Fig Fb: In this case, the locations of the node having a large k^in^ are mainly in the core far away from those of the nodes having a large k^out^ within the shell. So, if r^in-in^ (or r^out-out^) is high, there is a high probability that the locations of the connected nodes would be close to each other; thus, the number of intra-subdivisional connections (N_cc_ and N_ss_) would be large, while that of inter-subdivisional connections (N_sc_ and N_cs_) would be relatively smaller. On the other hand, a high level of r^in-out^ means that a postsynaptic node having a large k^in^ (mostly located in the core) is more likely to connect to a presynaptic node having a large k^out^ (mostly located in the shell); consequently, the number of connections between the two subdivisions (N_sc_ and N_cs_) will become large at the expense of N_cc_ and N_ss_. Even for this case of negative k^in^ - k^out^ correlation, N_cc_ is much larger than N_ss_ (not shown), therefore the role of N_cc_ is more pronounced over that of N_ss_.

Next, we consider the colormap of the 3^rd^ row – 1^st^ column (k^total^ referenced core-dense case) of S7b. For this case, there is a high probability that nodes with a large (small) k^in^ and those with a large (small) k^out^ are in the core (shell). Therefore, r^in-out^ (or r^out-in^) is positively, while r^in-in^ (or r^out-out^) is negatively, correlated with N_cc_. So, the situation is somewhat opposite to the map of the 1^st^ row – 1^st^ column of Fig Fb described just above. Previously, we showed that for the negatively correlated k^in^ - k^out^, r^out-out^ is positively correlated with r^in-in^ but negatively correlated with r^in-out^ and r^out-in^ (see Fig Fg). So, all the results are consistent. Concerning the correlation maps shown in Fig F, so far we have discussed their origins only for three cases. As for the other cases, a similar set of arguments can be applied for the elucidation of their origins; thus, we will not discuss them all one by one. After all, these computed correlation maps would be only a useful reference guide for elucidating the successful network models of the SCN.

**
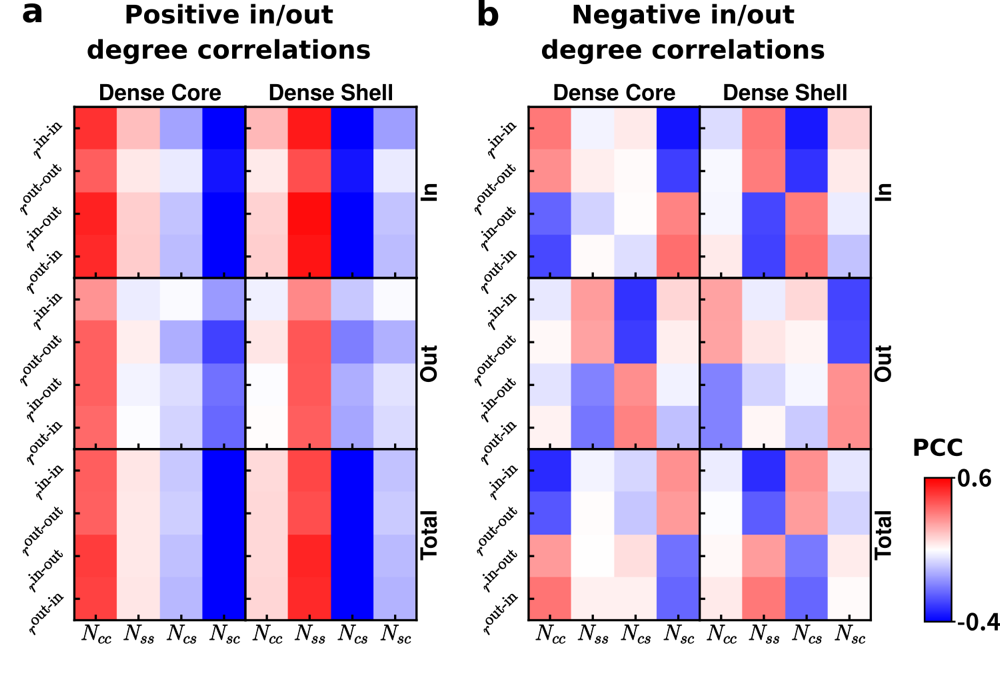
**

**Fig F. Colormaps showing the level of pairwise Pearson correlation (PCC) between the numbers of 4 different types of edge connection and 4 different types of assortativity.** (a) and (b) are for two different cases when k^in^ is positively and negatively correlated with k^out^, respectively. The ‘Dense Core’ and ‘Dense Shell’ refer to the two different spatial mappings discussed in Fig 4a (in the main text). The labels ‘In’, ‘Out’, and ‘Total’ refer to the 3 different cases of using k^in^, k^out^ or k^total^ as a reference variable used for the 2D spatial arrangement of nodes. This correlation maps are based on the pool of 25,140 model networks (see Table 1 in the main text).

**8. Weak dependence of some key SCN phase dynamic as well as network graph-theoretical measures on the node density**

As we have claimed in the main text, neither a 20% increase nor a 20% decrease in the number of cells changes key characteristics of the chosen model SCN networks as shown in Fig G.


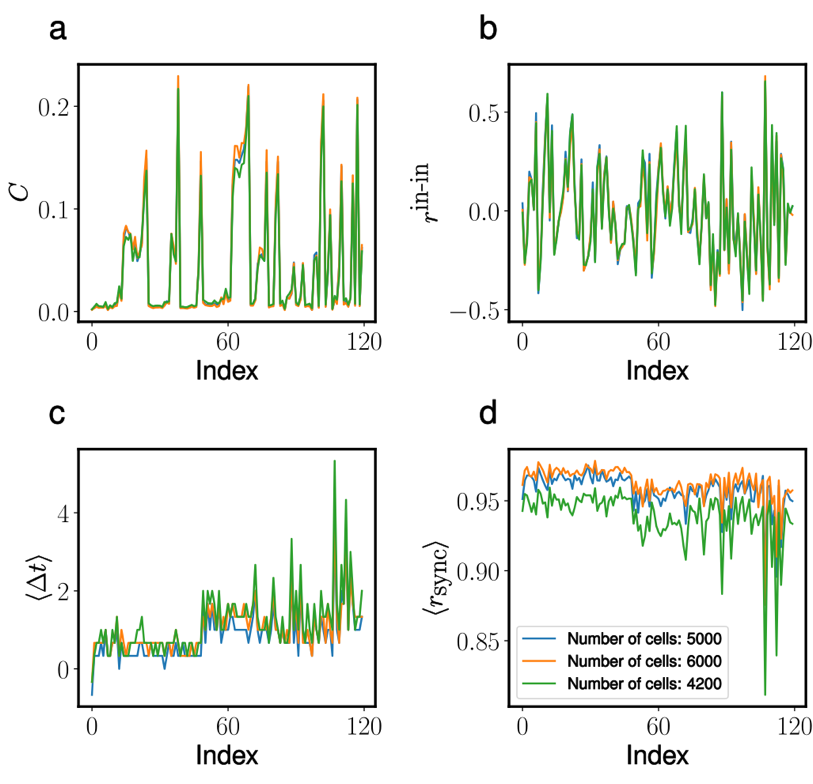


**Fig G. Network morphology and phase wave properties are not sensitive on node density.** Two graph-theoretical measures of network morphology (a: clustering coefficient $C$; b: assortativity $r^{in-in}$) and two different measures of phase wave dynamic properties (c: average core-shell phase difference $<\Delta t>$; d: average phase synchrony $<r_{sync}>$) for 120 different networks. The 120 networks are uniformly sampled from the final pool of 1,084 SCN model networks and different colored lines represent a different number of nodes forming the system.
