# Supplementary figures and images for: Deciphering clock cell network morphology within the biological master clock, suprachiasmatic nucleus: From the perspective of circadian wave dynamics
Source: PLoS Comput Biol. 2022 Jun 6;18(6):e1010213. doi: 10.1371/journal.pcbi.1010213 (PMC9203024; doi:10.1371/journal.pcbi.1010213)

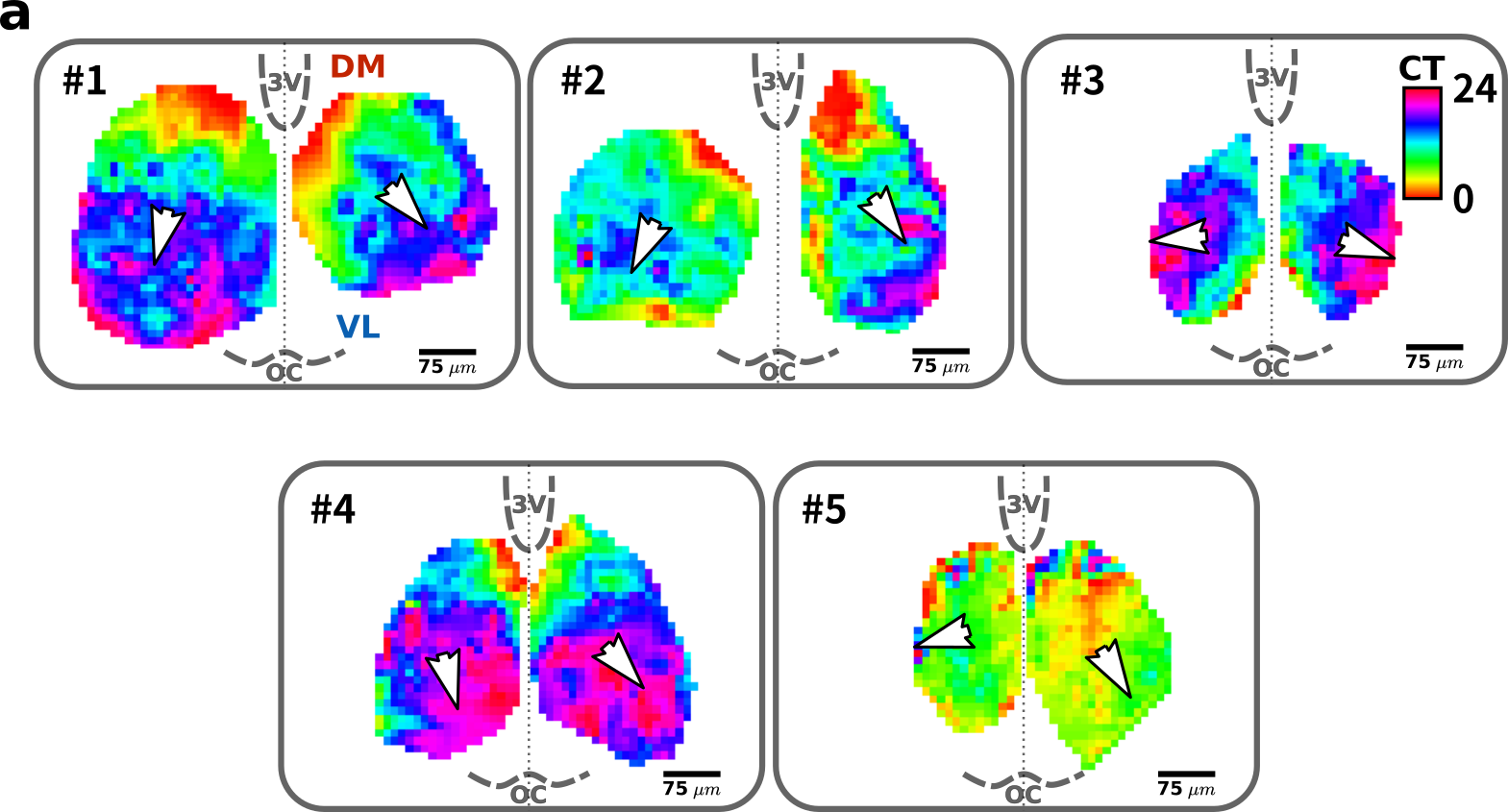

Supplement: S1 Fig — 2D snapshots of circadian phase maps (all images are reoriented such that the midline of the third ventricle (3V) aligns along the y-axis). The direction of each white arrowhead depicts the average instantaneous direction of phase wave propagation. Different phase maps correspond to a different organotypic ex vivo mouse SCN (coronal section) culture, and they are taken at different days (and hours)-in-vitro (DIV). The exact geophysical times when the phase maps were acquired are unknown. The SCN slice cultures are quasi-3D (with a typical thickness of 100 ~ 150 μm); therefore, shown are 2D projection images of 3D samples. The frame #1 corresponds to Fig 1A. (The original PER2::LUC signal data were shared at https://github.com/johnabel/scn-resynchronization-data-2016). (TIFF) [file pcbi.1010213.s002.tiff]

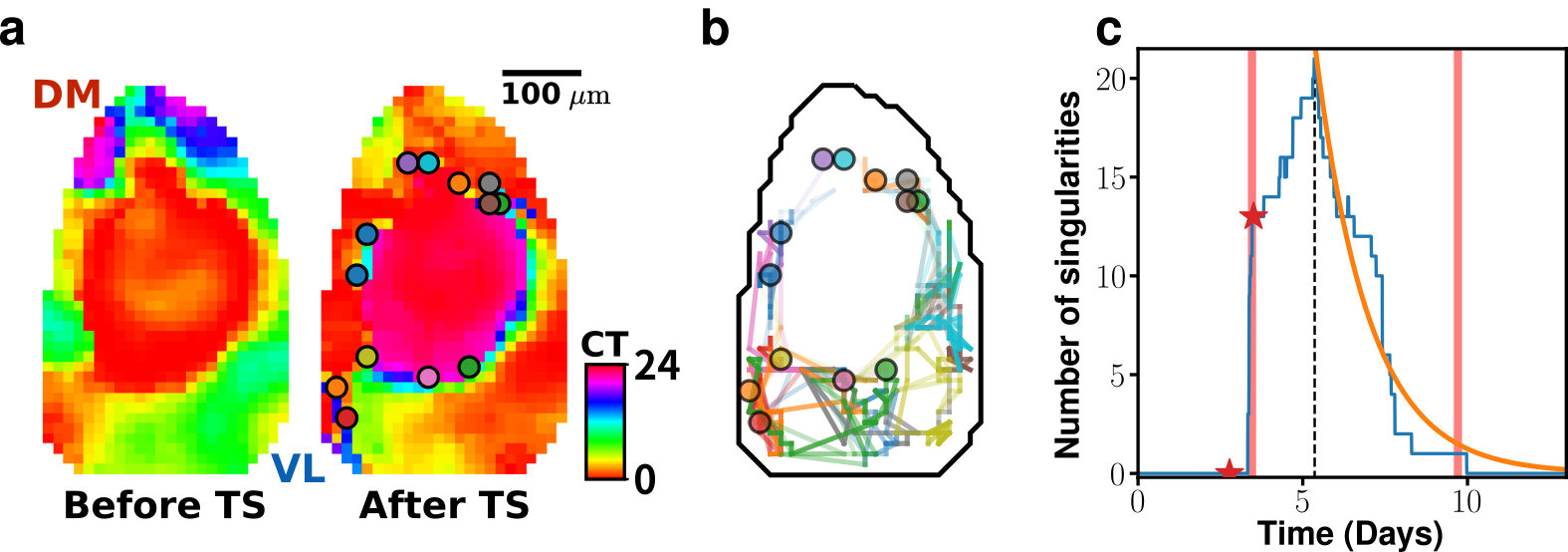

Supplement: S2 Fig — (a) Snapshot circadian PER2::LUC phase maps, just before (left) and 4 hr after (right) a temperature shock (TS) was delivered. The colored dots in (a) mark the positions of all phase singularities at the star-marked time point in (c). Phase singularities are identified by employing an Iyer-Gray method [67]. (b) Temporal traces of all phase singularities, from their births to deaths. A Crocker-Grier linking algorithm [68] is used for linking a set of different singularities identified at different time points. (c) Temporal evolution of the total number of phase singularities. The lifetime of the perturbation-induced phase singularities is about 3 days. The first (second) temperature shock pulse is given at t = 3.3 (9.6) days; the time points of the two heat shocks are marked by two red vertical lines. We point out that a timely perturbation is very critical for the creation of phase singularities: For instance, the second perturbation given approximately at day 9.6 has failed to produce any phase singularities. [Our (BJ and KJL) earlier experimental data published in Ref. [9] is used for this new analysis.] (TIFF) [file pcbi.1010213.s003.tiff]

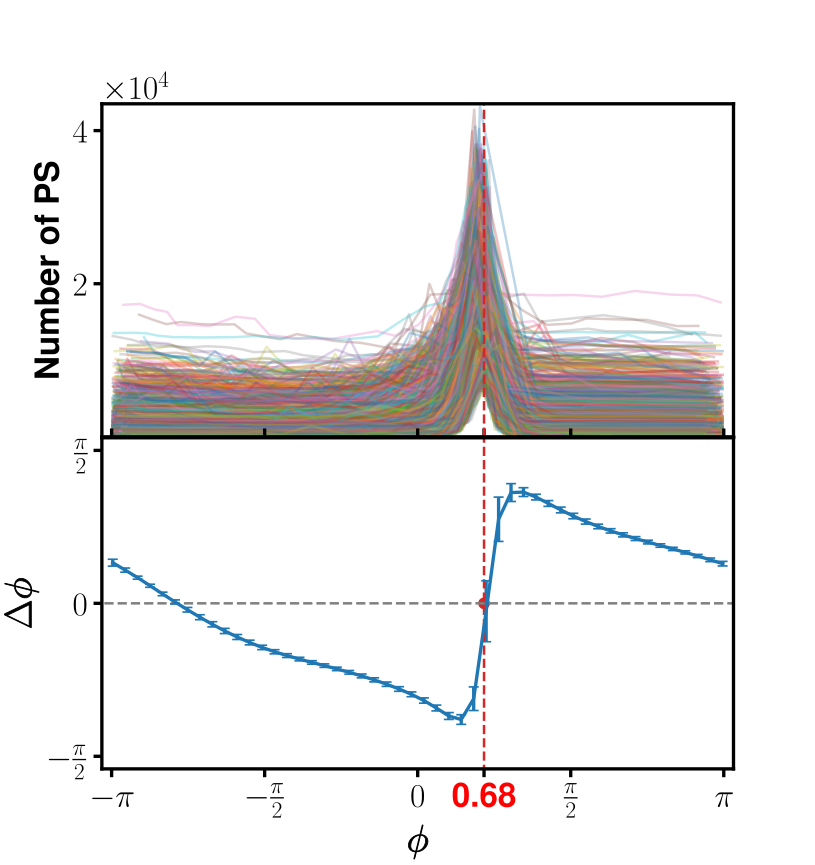

Supplement: S3 Fig — As for the perturbation, 0.7×ri where ri=xi2+yi2, is subtracted from every node I at a single circadian instance in order to mimic the decrease in the level of PER2::LUC subject to a temperature pulse shock [9]. The top frame includes 3,500 lines, each of which corresponds to a randomly selected network (having rsync>0.9) of our SCN model archive. Note that for all cases Npsmax peaks around the same unstable fixed point (marked by a red dot) of the PRC. Δϕ is the amount of mean (over 3,500 cases) phase shift due to the homogeneous perturbation and the error bars represent the standard deviation of Δϕ. (TIFF) [file pcbi.1010213.s004.tiff]

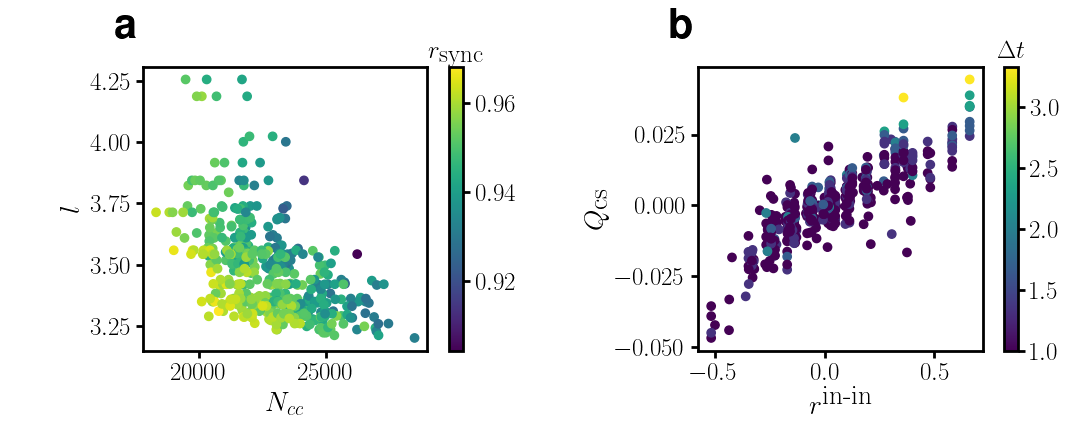

Supplement: S4 Fig — The level of phase synchrony in (a) and average phase difference between the core and shell (b) as a function of various network properties. Qcs is the modularity score based on two modules (core and shell). (TIFF) [file pcbi.1010213.s005.tiff]

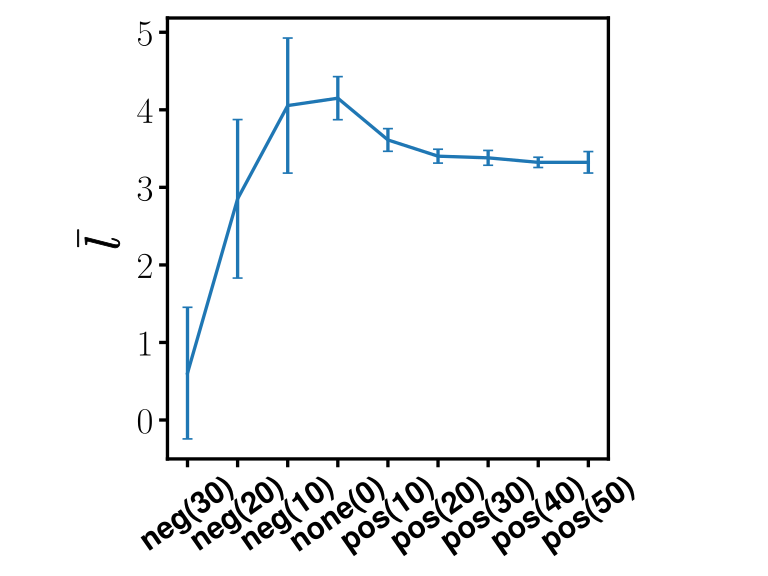

Supplement: S5 Fig — Note that l¯ does not change much for networks having a positive indegree-outdegree relation. (TIFF) [file pcbi.1010213.s006.tiff]
